# A technical survey on practical applications and guidelines for IoT sensors in precision agriculture and viticulture

**DOI:** 10.1038/s41598-024-80924-y

**Published:** 2024-11-30

**Authors:** David Pascoal, Nuno Silva, Telmo Adão, Rui Diogo Lopes, Emanuel Peres, Raul Morais

**Affiliations:** 1 Department of Engineering, School of Sciences and Technology, University of Trás-os-Montes e Alto Douro, Quinta de Prados – Folhadela, 5000-801 Vila Real, Portugal; 2 Centre for the Research and Technology of Agro-Environmental and Biological Sciences, University of Trás-os-Montes e Alto Douro, 5000-801 Vila Real, Portugal; 3 ALGORITMI Research Center/LASI, University of Minho, Alameda da Universidade, 4800-058 Braga, Guimarães, Portugal; 4 Institute for Innovation, Capacity Building and Sustainability of Agri-Food Production, University of Trás-os-Montes e Alto Douro, 5000-801 Vila Real, Portugal

**Keywords:** Precision agriculture, Precision viticulture, Internet-of-Things, IoT, Sensor networks, Smart irrigation, Smart fertilization, Pests and diseases detection, Vegetation cover monitoring and mapping, Climatic monitoring, Sensor installation best-practices, Crop productivity, Crop sustainability, Climate sciences, Environmental sciences, Engineering

## Abstract

Climate change pose significant challenges to modern agriculture management systems, threatening food production and security. Therefore, tackling its effects has never been so imperative to attain sustainable food access and nutrition worldwide. In the case of viticulture, besides jeopardizing grape production, climate change has severe impact in quality, which has becoming more challenging to manage, due to the increasingly frequent fungal contamination, with consequences for relevant quality parameters such as the aromatic profiles of grapes and wines and their phenolic compounds. This has been leading to a reconfiguration of the wine industry geostrategic landscape and economy dynamics, particularly in Southern Europe. To address these and other emerging challenges, in-field deployable proximity-based precision technologies have been enabling real-time monitoring of crops ecosystems, including climate, soil and plants, by performing relevant data gathering and storage, paving the way for advanced decision support under the Internet of Things (IoT) paradigm. This paper explores the integration of agronomic and technological knowledge, emphasizing the proper selection of IoT-capable sensors for viticulture, while considering more general ones from agriculture to fill gaps when specialized options are unavailable. Moreover, advisable practices for sensor installation are provided, according to respective types, data acquisition capabilities and applicability.

## Introduction

Agriculture, covering nearly 40% of Earth’s land surface, has been a primary driver of the Anthropocene era, marked by human activities profoundly impacting natural systems, but, mostly, having a crucial role in global food security, while technological innovations have been progressively allowing to boost productivity^1^. Within the agricultural context, grapevines are among the most important crops, as can be inferred from *Organisation Internationale de la Vigne et du Vin* (OIV) official reports^2^. Some of the relevant numbers of these reports are highlighted as follows:

- There are more than 7 million hectares of planted vineyard area, used to produce not only table grapes, but also other grape derivatives, such as wine, juice, and raisins;
- Wine production has been stabilizing in the 260 million hectoliters per year, roughly;
- In 2022, the estimations for wine consumption were around 232 million hectoliters;
- Wine exportation in 2022 reached a record value of 37.6 billion euros.

However, climate change, showing no signs of interruption or slowdown^3,4^, has been leading to challenges underlying agricultural practices. Their impacts on agricultural productivity can be classified in two categories: direct and indirect. While the former refers to variations in temperature and rainfall distribution affecting phenological cycles regular chronology^3^, the latter is mostly related with the influence over different species’ behavior and dispersion, such as pollinators, invasive species, pests, and disease vectors^3^, which end up shaping crops development. These aspects not only will impact the way the soil is managed, as it will greatly reconfigure food access to meet worldwide needs, either in terms of quantity, nutrients and safety^5,6^.

Similarly, viticulture has been also affected by the potential harmful effects of climate change in crops production and quality^7,8^, due to the occurrence of violent storms or accumulated heat stress, limited access to water and reduced rainfall leading to increased evapotranspiration rates during the developmental stages. Moreover, global warming is favoring the development of specific grapes fungi that release great concentrations of mycotoxins that negatively impact wine’s quality^9^. Additionally, the aromatic potential, quality, and sensory perception of wines is also changing due to a more restrictive water regime, which along with temperature effects, alters the metabolism of the fruit and in some cases can lead to defective maturation. Conversely, in cases where complete maturation is reached, decreased wines’ vegetal characteristics has been observed, while floral and fruity aromas are intensified. Notwithstanding, wines’ acidity is affected, as well as their aging potential^10,11^. Such climate behaviors are also geographically reconfiguring viticulture productivity landscape, with regional influence over the Western and Central Europe^8^.

Given the aforementioned challenges in the agricultural sector, including viticulture, technological solutions are key to more effectively monitoring and managing crop conditions. More specifically, cost-effective and mature data gathering technologies have been developed and deployed in support of farming practices, contributing for an increased sustainability in agriculture/viticulture. Due to the capacity of electronically sensing the several factors affecting soil, climate, and crops, and storing such observations into dedicated data lakes, avenues for data analytics and decision support are paved, aiming at relevant events anticipation—related but not confined to abnormal climate occurrences—,and resources optimization (e.g. water and phytosanitary products administration). This concept has been successfully materialized through wireless sensor networks (WSNs) operating under an Internet-of-Things (IoT) paradigm^12–16^.

**Fig. 1:**
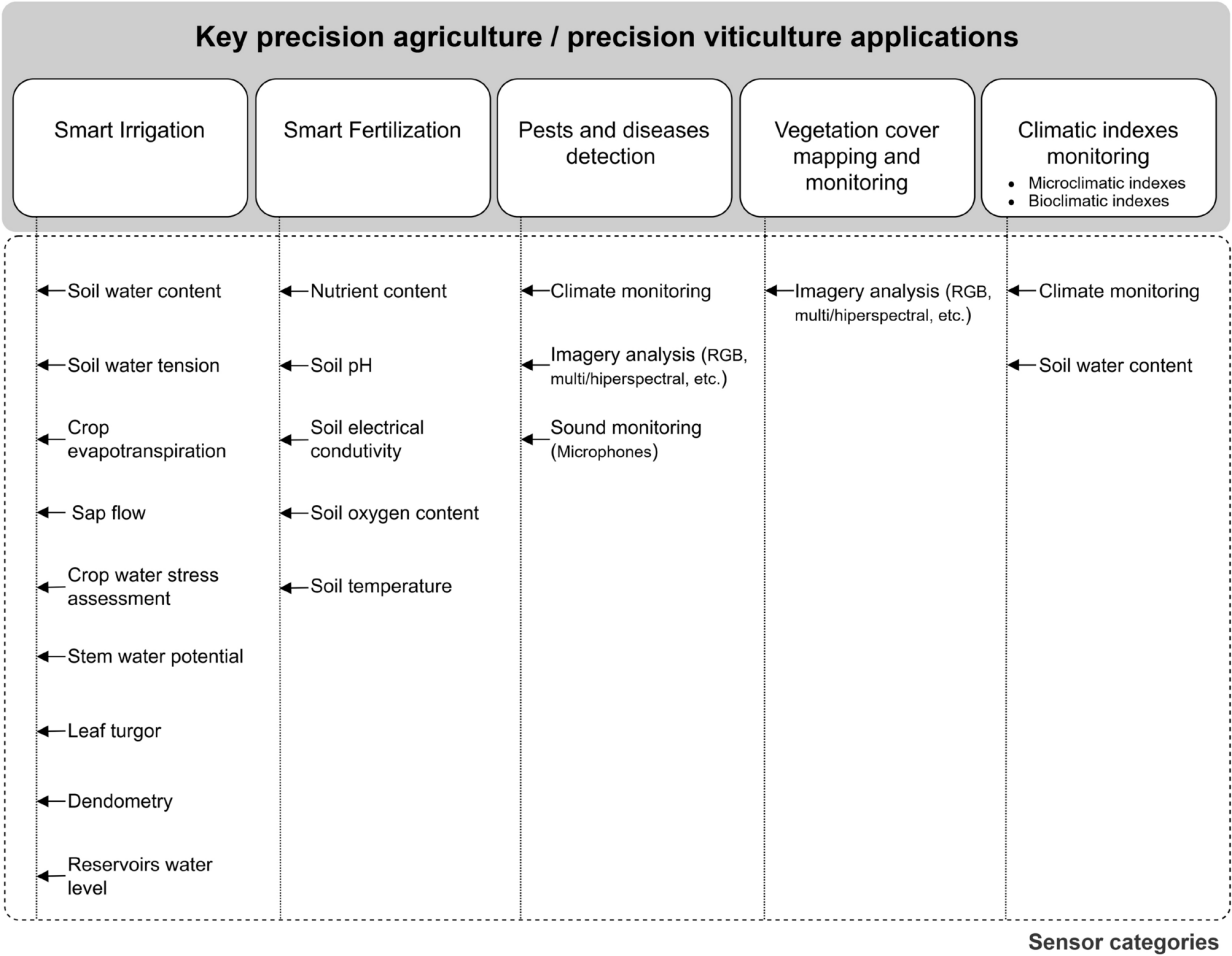
Categories of PA/PV stationary close-range IoT-capable sensors for field installation: a taxonomy-based proposal.

Having in mind the set of previously addressed topics and issues, in this paper, a technical-scientific literature-based survey is presented, focusing on close-range IoT-capable sensors for performing agronomic-oriented monitoring operations within the scope of precision agriculture (PA) and their specific applications in precision viticulture (PV), considering the categorization depicted in Fig. 1. Based in such categorization, concrete examples of IoT-capable sensors are proposed as baseline references for informed selection when setting up PA/PV monitoring environments, regardless of manufacturer or brand. More specifically, starting by focusing the context of viticulture, parameters measured by commercially available IoT sensors are identified, and, for those cases where direct solutions are unavailable, more general devices adapted from agriculture are proposed. Furthermore, sensors applicability, respective advantages/disadvantages and best utilization practices are encompassed, with the aim of providing a set of guidelines for farmers interested in implementing objective-oriented solutions resorting to specific context-sensitive electronic devices. Therefore, this document also intends to guide professionals/practitioners in selecting sensor types according to their specific needs (e.g., irrigation, fertilization, etc.). Outside the scope 3 of this paper are remote sensing systems based on satellites, aircraft, and unmanned aerial vehicles, instrumented terrestrial vehicles of any kind (e.g. tractors), as well as robotics, mobile platforms and akin technologies.

Considering the aforementioned topics under analysis, the following combination of contributions outlines the innovation encompassed by this work:

- Comprehensive survey of close-range sensing approaches: a thorough examination of the main approaches for monitoring parameters crucial to agricultural and viticultural crop development is presented. This comprehensive survey integrates both agronomic knowledge and sensor technologies, contributing to more informed and precise farming management practices.
- Guided sensor selection support: purpose-driven orientations to carry out IoT-capable sensors selection are provided. Association between monitoring goals with off-the-shelf sensors are highlighted to aid professionals in setting up agricultural/viticultural-aware digital-based environments.
- Emphasis on relevant advantages/disadvantages and best-practices for sensor installation: pros and cons of each sensor type, as well as installation recommendations and precautions are encompassed, ensuring data gathering operational effectiveness. This focus on proper installation methods is essential for achieving reliable and scalable solutions in real-world farming environments.

In terms of structure, this paper is organized as follows: the “IoT-based field sensors for PA/PV” section describes the areas of agronomy that benefit from IoT, detailing the most suited sensor types for the different monitoring goals, respective data acquisition capabilities and relevant advantages/disadvantages; the identified types of agricultural application applying specific examples of manufactured IoT sensors are then addressed in the section with the title “PA/PV applications, techniques, and measurements: a quick guide towards the selection of IoT-capable sensors”, categorizing them according to their use, whenever applicable; next, in the section dedicated to “IoT-capable sensors installation: a practical guide”, relevant procedures for setting up IoT sensors in the field are addressed, along with considerations regarding recommendations and precautions, as well as a few insights on sensor fusion strategies; finally, a critical analysis is conducted in the “Discussion and conclusions” section, in which final remarks are also presented.

## IoT-based field sensors for PA/PV

As an increasingly popular multi-purpose paradigm, IoT can be characterized by the use of electronic devices, sensors, actuators, and communication components integrated directly in a technological infrastructure. This paradigm has been responsible for accelerating the digital transition in the agriculture/forestry fields, wherein data gathering techniques have been playing a central role in the tracking of the direct and indirect factors that influence crops behavior. In that way, IoT devices allow farmers to perform *in situ* monitoring and control of critical and/or essential factors related to, for example, irrigation, fertilization, crops development, among other environmental conditions. In complement, vitally supported by its sensing capabilities, IoT can extend features to automatize tasks in a time-effective manner, such as the triggering of irrigation systems^17^, based on rule-based alarm handling, data analysis, machine-based recommendations/decisions, etc.. Indeed, the amount of data that IoT-based sensors can produce from contextual monitoring enables advanced and automated decision support systems, empowering farmers to carry out informed interventions in the field and apply the correct resources (e.g. water, fertilizers, phytopharmaceutical products) in roughly adequate quantities, preventing undesirable wastes and reducing the ecological footprint. When layered with an operational frontend, IoT becomes interactive and, therefore, tangible for human users, through graphical interfaces and respective information visualization strategies (e.g. dashboards), touch screens, and voice command systems, etc.^18^, as demonstrated in previous works^19^.

In line with the previous contextualization, a survey on close-range field sensors for IoT-based precision viticulture, is presented, considering a collection of fundamental applications that have been categorized based on the consulted literature:

- Smart irrigation,
- Smart fertilization,
- Pests and diseases detection,
- Vegetation cover monitoring and mapping,
- Climatic indices monitoring.

To cover gaps in precision viticulture IoT sensors, alternatives commonly used in broader agricultural applications are considered.

### Smart irrigation

Excessive irrigation raises production costs due to unnecessary water and energy use, while also causing soil erosion, nutrient leaching, and increased fertilization needs. Over-irrigation spreads contaminants like fertilizers, worsening diseases, fostering anaerobic soil conditions, and complicating field operations^20^. Conversely, insufficient irrigation hampers crop development, reducing transpiration, photosynthesis, and ultimately grape yield and quality^20,21^. However, insufficient irrigation differs from deficit irrigation, as the latter is a technique used in the final grapevine stages to enhance fruit quality for winemaking^21^.

Currently, there are several technologies that support the assessment of water purport/stress, either focusing soil or plans, allowing to effectively implement irrigation management strategies with the goal of optimizing hydrological resources usage, reducing, simultaneously, production costs while ensuring crops yield and quality^21,22^. More specifically, common irrigation management techniques leverage from assessing water content regarding soil^23^, plants^24^, and evapotranspiration-related processes^24^, harnessing from the panoply of agricultural biophysical mechanisms that can be used as signaling pathways to inspect vegetation’s intrinsic conditions associated to hydration^25^. Such techniques are addressed as follows.

#### Soil water content

The determination of soil water content, at different depths, and its transit over a certain period of time is one of the most commonly used methods for irrigation cycle management^23,26^. Its purpose revolves around determining when the soil water content reaches a critical limit. In response to such occurrence, an irrigation campaign should be carried out to restore the water that the soil has lost by plant transpiration or other ways^27^.

#### Soil matric potential

The determination of soil matric potential and its evolution over time is another method for irrigation management, being tensiometers the most commonly used method for calculating soil matric potential. Despite the possibility of adapting tensiometers to connect to low-cost microcontrollers shown in some of the literature^28,29^, currently, other types of IoT sensors that determine the soil electrical resistance and calculate indirect soil matric potential are used, with the advantage of being simpler to assemble^30^ and enabling remote data reporting based in a server-client architecture^31^.

#### Crop evapotranspiration

Evapotranspiration refers to a process that allows the determination of plant water needs through the quantification of water loss in a given environment. It can be used to assess the effect of the climatic conditions around the field where the crop of interest is installed, based on the following biophysical measurable indicators: i) water evaporation from the soil; and ii) water transpiration through plant’s stomata. The proportion of evaporation and transpiration as partial components of evapotranspiration varies according to the plant’s phenological stage, more specifically, depending on the leaf area per adjacent soil surface unit. During the initial stages of the vegetative cycle, soil water evaporation is what most contributes to evapotranspiration. When the plant’s leaf area is fully developed, more than 90% of evapotranspiration is due to transpiration. The crop evapotranspiration ($ET_{\textrm{c}}$) is determined as shown in Eq. 1:

1 $$\begin{aligned} ET_{\textrm{c}} = ET_{0} \cdot K_{\textrm{c}} \end{aligned}$$

where $ET_{\textrm{0}}$ stands for reference evapotranspiration—using meteorological data obtained throughout the day, namely air temperature and relative humidity, wind speed, and solar radiation—and $K_{\textrm{c}}$ regards to the cultural coefficient, which changes according to the culture being analyzed and to the plant’s development stage^21,32^. Climate variable monitoring can be done through low-cost sensors with connectivity capabilities^33^.

#### Sap flow

Sap flow sensors are commonly built over heat dissipation probes and temperature sensors strategically placed on the trees’ trunks—also, applicable to grapevines—, which analyze the dissipation of heat by the sap. The main methods of determining sap flow through thermal dissipation are based on two techniques: heat dissipation (introduced by Granier) and heat balance^34^. However, there are studies stressing out precision issues related to the readings made by this class of IoT sensors^35–37^. On the one hand, Granier’s heat dissipation method is widely used because of its simplicity. It measures heat dissipation given by the temperature difference between a pair of probes implanted in the trunk in relation to sap flow^38^. On the other hand, the heat balance method determines how much heat is needed to keep a steady temperature difference along a section of the trunk^39^. Although both methods are the most common^34^, another approach based on heat pulse is gaining relevance due to the higher accuracy that it currently presents. It operated through the emission of a heat pulse is to the plant’s trunk, from which spreading time is measured^40^. For a long time, the sensors used for heat balance and dissipation methods were considered more invasive for plants. However, strategies more integrity-friendly have been made available, such as microneedles^34,41^ and even devices that fit around the plant’s trunk without requiring intrusion^40^.

A relevant aspect that should be taken into account regarding the sap flow sensors is that they alone can only indicate the speed of heat displacements. To calculate the transpiration rate, complementary data from the plant’s biophysics is required, which often consists in using destructive methods^42^. Also, the determination of water stress cannot be directly inferred due to the strong influence of VPD (Vapor Pressure Deficit) on the measurements^43,44^. These factors are of important consideration to properly help farmers in building an overview of the plant’s water consumption patterns and, therefore, assisting them in the optimization of irrigation procedures scheduling^38^.

In viticulture, sap flow can also be useful for winemakers to achieve enological goals that add value to the grapes, such as enhancing sugar content and the accumulation of phenolic compounds contributing to the color of the grape clusters^45^.

#### Crop water stress index

Water loss through transpiration is a process that enables the plant to regulate its leafs’ temperature, as transpiration cools down the leaf surface by releasing the latent heat in contained water. When the plant is in a state of hydric comfort, its transpiration is stimulated and the canopy temperature is regulated. With the decreasing of soil moisture, the transpiration rate tends to be reduced while the temperature of the vegetation cover increases, making the plant’s hydric conditions to move away from the comfortable status^25^.

The crop water stress index (CWSI) can be used to determine the plant water status by subtracting the canopy temperature (Tc) from the air temperature around the plant (Ta). To determine the plant water status, two reference points are needed—hydric stress condition (UL) and hydric comfort condition (LL)—, which will serve as a comparison term for the Tc-Ta value obtained at the checking moment (M)^46^. The CWSI, found in works such as^46,47^, is calculated using Eq. 2:

2 $$\begin{aligned} CWSI = \frac{{(T_{\textrm{c}} - T_{\textrm{a}})_{\textrm{M}} - (T_{\textrm{c}} - T_{\textrm{a}})_{\textrm{LL}}}}{{(T_{\textrm{c}} - T_{\textrm{a}})_{\textrm{UL}} - (T_{\textrm{c}} - T_{\textrm{a}})_{\textrm{LL}}}} \end{aligned}$$

The reference point $(T_{\textrm{c}} - T_{\textrm{a}})_{\textrm{UL}}$ describes the temperature of a non-transpiring leaf with fully closed stomata. Moreover, $(T_{\textrm{c}} - T_{\textrm{a}})_{\textrm{LL}}$ represents the temperature of a leaf with fully open stomata – i.e., normal transpiration^48^. There are several ways to determine the reference points associated to $(T_{\textrm{c}} - T_{\textrm{a}})_{\textrm{UL}}$ and $(T_{\textrm{c}} - T_{\textrm{a}})_{\textrm{LL}}$, also known as $T_{dry}$ and $T_{wet}$, respectively. Relevant approaches are listed in Table 1.

**Table 1:** Approaches to determine $(T_{\textrm{c}} - T_{\textrm{a}})_{\textrm{UL}}$ ($T_{dry}$) and $(T_{\textrm{c}} - T_{\textrm{a}})_{\textrm{LL}}$ ($T_{wet}$).

Approach/Reference	$(T_{\textrm{c}} - T_{\textrm{a}})_{\textrm{UL}}$ ($T_{dry}$) determination procedure	$(T_{\textrm{c}} - T_{\textrm{a}})_{\textrm{LL}}$ ($T_{wet}$) determination procedure
Vaseline and water spray^49^	Temperature measured after covering the leaves with vaseline.	Temperature measured after spraying the leaves with a water-containing spray.
Air temperature and water spray^48^	Air temperature measured at a given moment +3 °C.	Temperature measured after spraying the leaves with a water-containing spray.
Canopy temperature in severe conditions and water spray^48^	Canopy temperature measured under severe water stress conditions.	Temperature measured after spraying the leaves with a water-containing spray.
Air temperature and good hydration conditions^48^	Air temperature measured at a given moment +3 °C.	Canopy temperature measured under good hydration conditions.
3 °C rule and good hydration conditions^48^	3 °C.	Temperature difference between the air and well-watered canopy.
Combined equations^50^	Combination of equations that correlate $T_{c} - T_{a}$ with the air VPD, net radiation, and both aerodynamic and crop resistances.	

The CWSI results range from 0 to 1, wherein the higher are the values, the greater are the levels of water stress in the plant^51,52^.

The use of infrared (IR) cameras, which capture thermal-sensitive images, is becoming increasingly common in agricultural applications. With the help of computer software, these images can be processed to determine the average temperature in a specific area, such as the temperature of the plant canopy. Additionally, the temperature distribution can be represented by different coloring scales/levels over the image, allowing detailed analysis of various sections such as ground, shaded areas, and areas highly exposed to the sun^53,54^. Some works using this kind of imagery devices can be found, as proposed by Ramos-Giraldo et al.^55^, who used a Raspberry Pi with a Sony IMX219 camera having stereo lenses and wide-angle IR/RGB-capable sensors, to monitor the hydric stress of corn and soybeans. Rather than resorting to any particular image index, they determined canopy temperature ($T_{\textrm{c}}$) relying in the IR sensor raw data. The stationary ground-station equipment was placed on a support whose angle and height varied depending on the culture under analysis – 9 m above the ground and 90° of inclination for corn culture; 6 m above the ground oriented at 45° for soybean culture.

#### Stem water potential

Stem water potential is a standard method for assessing the grapevines’ water status, which provides a direct measurement of water tension inside the plant^56,57^, by combining multiple factors related to the effects of soil, atmosphere, and the the biophysics of the plant itself. However and despite the effectiveness of the technique, some issues are worthy to highlight. More specifically, it is a time-consuming technique, which harms the plant, and, to work properly, requires a special tool known as Scholander pressure chamber^58^. Additionally, the water potential measured before dawn and recognized as a reliable indicator of water status in grapevines^59^, requires nocturne visits to the field.

Recently, advances in sensor technology have led to the development of microtensiometers that continuously measure water tension in the woody tissue of trees^60^. These devices show how plant water status can change throughout the day^58^. When integrated with IoT devices, these measurements become automatic, continuous, and direct, enabling constant monitoring of the plant water status^61,62^.

#### Leaf Turgor

Also used to monitor the water status of plants, the turgor sensor consists of a pressure chip embedded in a gel portion placed inside a plastic chamber. This chamber is surrounded by a metallic ring which, along with a counteracting magnet, is attached to the plant’s leaf. As the plant’s water status changes, the leaf thickness increases or decreases, being these variations captured by this type of sensors^63^.

Leaf turgor reaches its peak during the day and decreases at night. When the plant is in a state of water comfort, the turgor sensor signal varies regularly. Under water stress, turgor may drop—most likely, during the day—, indicating difficulties in water absorption, possibly leading to leave degeneration (wilting). In the most severe cases, the signal may be lost completely, even after reintroducing water. At this point, the plant may not be able to recover. Therefore, monitoring closely the turgor signal behavior is essential for quickly detecting water stress and time-effectively acting to restore the plant’s health^64^.

#### Dendrometry

The plant water status is determined by the amount of water the plant can absorb. The higher the water absorption, the higher the transpiration rate of the plant. Therefore, a correlation can be made between the transpiration rate of the plant and its water status^65^.

When a plant dissipates water through transpiration, a tension is generated in the leaves xylem that affects the plant holistically. This causes the organs to release their water reserves—stored during the night—for the leaves evaporative surface. Through this process, the plant can quickly respond to atmospheric changes demanding for evaporation, as water absorption by the roots takes some time to initiate^66^.

As the organs of the plant are capable of daily storing and releasing to the leaves, their diameter changes throughout the day. To measure these biophysical manifestations, dendrometers are ideal instruments, allowing for the tracking of water movement within the plant as it enters in and exits from its organism, therefore, providing clues of plant water status^67^. To obtain greater precision in the water needs and plants’ response to environmental changes, the work^24^ recommends to combine dendrometry techniques and sap flow sensing approaches, namely for application in grapevines species.

Dendrometers are categorized into two main types: trunk dendrometers and fruit dendrometers^68^. The former measures daily variations in trunk diameter, which are directly influenced by soil water availability and atmospheric evaporative demand^66,69^. Under suitable conditions of water availability, there is a greater amplitude in the fluctuation of trunk diameter throughout the day. However, when the plant experiences water stress, this daily variation tends to be smaller.^69^. As for the latter category, it focus on the fruit growth rate (FGR). A significant decrease in FGR can indicate that fruit growth is being limited. This growth limitation may be associated with a water deficit, which could suggest that the plant might be experiencing water stress^68^. However, due to the sensitive biophysics of grape clusters, the FGR-based method does not seem to be applicable.

Currently, several types of dendrometers are available, as listed bellow:

- Band-type dendrometers: these are instruments that use a flexible band to monitor variations in the perimeter of a plant’s trunk, stem, or branch. This band is connected to a linear potentiometer, which converts physical changes into measurable electrical signals. The operating principle is based on the expansion and contraction of the stem or fruit diameter where the dendrometer is installed: when the diameter increases in volume, the band expands, and when it decreases, the band contracts. These changes are detected by the potentiometer, generating data that reflect changes in the plant’s diameter over time^70^;
- Linear potentiometer-based dendrometers: this kind of dendrometers are also instruments used to measure variations in radial or diametrical growth of a tree trunk, but resorting to a linear potentiometer or sensor externally fixed to the trunk through a rod. This device detects changes in stem volume, converting them into electrical signals. Changes in the output electrical voltage reflect the trunk’s growth pattern, allowing continuous and non-invasive monitoring of tree development. This technique provides valuable information about plant health and its response to environmental conditions, being particularly useful in forestry and agricultural studies^71^;
- Infrared and optical dendrometers: the optoelectronic dendrometer resort to infrared light, offering high resolution (up to 600 nm). Although,it has a limited measurement range and requires bulky equipment. Alternatively, the optical dendrometer, based on fiber optics, can measure up to 5 mm with a resolution of 5 µm, but also has measuring range restrictions. Moreover, its complex structure may require installation by specialized technicians. Both types, although innovative, still face challenges in terms of practicality and widespread applicability in the field^72^.

#### Water reservoirs instrumentation

To maximize the efficiency of water use, it is essential to consider the monitoring of reservoirs, enabling to accurately budget the available hydric resources for agricultural purposes, therefore contributing for a better management of farming activities^73,74^. The same principle can be replicated to multiple reservoirs, wherein an independent hydric resources management per station must be carried out, while keeping a holistic perception of agricultural needs in a given context, for a proper water administration scheduling. Pumping stations between the various reservoirs is one of the options to extract an deliver water to the targeted crops, as proposed in^75^.

#### Comparative analysis of smart irrigation methods: advantages and disadvantages

Methods used in smart irrigation systems are several and varied. In Table 2, main advantages and disadvantages of each analyzed method are highlighted. The aim is to provide a critical evaluation of the available options, as well as insights for informed decisions when it comes to select an irrigation approach, depending on the specific goal and context.

**Table 2:** Advantages and disadvantages of smart irrigation-related methods.

Method	Advantages	Disadvantages	References
Soil water content	Simple to install Highly precise Various commercial systems are available Measurements can help to smoothly determine water needs Certain sensors, such as capacitance and time domain sensors can be integrated in automation system with relative ease.	Soil variability requires the use of numerous sensors It is challenging to choose a location that accurately represents the root zone Sensors do not typically assess the water status directly at the root surface, which is influenced by evaporative demand Data logger integration is expensive.	^76,77^
Soil matric potential	Simple to install Highly precise Various commercial systems are available.	Soil variability requires the use of numerous sensors It is challenging to choose a location that accurately represents the root zone Regular maintenance is required Salinity and temperature may affect sensors’ performance Sensors do not typically assess the water status directly at the root surface, which is influenced by evaporative demand.	^76,77^
Crop evapotranspiration	Simple to install Provides directly usable data to manage water.	Less precise than carrying out a direct measurement to the plant (e.g. through sap flow) Requires accurate local weather data Accurate crop coefficients are essential for evapotranspiration estimation, which is influenced by crop growth and root depth As it is prone to readings drifts (errors), regular calibration is necessary.	^76^
Sap flow	Highly responsive.	Provides only indirect estimates of changes in conductance, as flow heavily depends on atmospheric conditions.	^76^
CWSI	Scalable for large crop areas, particularly with imaging technology The most simple form of thermometers are inexpensive and portable Compatible with continuous monitoring purposes.	Requires sophisticated instrumentation and technical expertise Calibration is needed for each tree and to determine irrigation control thresholds.	^76^
Stem Water Potential	Measures the pressure component of water potential, essential for xylem water flow and cell functions like growth Technologies (e.g. microtensiometers) compatible with IoT, suitable for automation strategies, as well as for continuous and direct measurements are already available.	Instruments must be handled with care and are typically expensive.	^61,62,76^
Leaf Turgor	Effective in detecting daily turgor variations and water stress, providing clear readings under ideal conditions.	Can be inaccurate during severe water deficit and requires frequent sensor replacements anticipating physiological changes.	^64^
Dendrometry	Changes in tissue water content are simpler to measure and to automate, when compared to water potential sensors Commercial sensors that measure small-scale structural features are available.	Instrumentation is generally complex or expensive, which constitutes a challenge to scalability Integration involves issues related to environmental variability, calibration requirements, and data interpretability.	^76^
Water reservoirs instrumentation	Level sensors allow to optimize water use.	Susceptibility to external interference, such as objects, dust, and other environmental elements can compromise the accuracy of sensor.	^78,79^

### Smart fertilization

Fertilization is one of the fundamental activities to consider in any agricultural crop. Therefore, regular monitoring of the nutrient content, pH^80^ and soil conductivity^81^ allows for interventions to optimize the productive yield of plants^80,81^. By using specific sensors to that end, it is possible to continuously determine the variability of the soil in a quick and non-destructive way^82^ and in a cost-effectively manner comparatively to traditional approaches, such as, laboratory analysis.

#### Nutrient content

In agriculture, nutrients necessary for plants are divided into two main groups: macronutrients and micronutrients. The former are depleted more easily in the soil due to the higher consumption demands by the plant, which also plays a greater role in plants’ development^83^. This group also includes other components such as iron, zinc, manganese, chlorine, copper, nickel, boron, and molybdenum, which can be found in plant tissues in reduced proportions, but with relevant participation in its physiological and metabolic processes^84^. In turn, the main group of macronutrients is divided into two subgroups: (i) primary, which includes nitrogen, phosphorus, and potassium, often in deficit in the soil, in spite of being the most consumed macronutrients^85^; and (ii) secondary, which groups calcium, sulfur, and magnesium^85,86^, with a greater importance for the physiological functions and metabolic processes of plants, rather than impacting substantially in productivity, when compared to the former subgroup^86^.

Regarding the methods for determining nutrients content in soil, there are several that can be found in literature, including:

- Conventional laboratory analyses^87^;
- Analysis by near-infrared spectroscopy in the laboratory^88,89^;
- Optical sensors^90,91^;
- Electrochemical sensors^92^;
- Soil electrical conductivity measurement sensors^93^.

Conventional laboratory analyses of soil, although complex and expensive, have the advantage of precisely quantifying both macro and micronutrients present in samples^87^. Some disadvantages include logistics and delays associated, respectively, to the need of carefully cataloging samples and transporting them to the laboratory facilities and, also, to the delivery of results^94^. Besides, samples selection zones are prone to uncertainties^87–92,95^. This makes the process irregular, discontinuous, and conditional in terms of time and resources. Alternatives include optical and electrochemical sensors, which have been used for the assessment of macronutrient content^92,96–98^. While spectroscopy for soil fertilization assessment is not a new topic^99^, only in recent years such grade of devices have becoming more affordable, as it can be inferred from the work of Beek et al.^94^, in which multispectral sensors are applied for soil analyses, quantifying both macro and micronutrients, with the advantage of providing quickly and *in situ* results. At usually higher price rates, hyperspectral cameras^100,101^ can also be used directly in the field, automatically transmitting data to farmers without this intervention through IoT platforms connected to cloud servers, such as Amazon Web Services, Microsoft Azure, Google Cloud, and others^102^. As for electrical conductivity, there are sensors directly applicable to the soil, more specifically wherein plants’ roots predominate, capable of performing continuous nutritional measurements^93,103^. Also, specific reading endpoints for measuring nitrogen (N), phosphorus (P), and potassium (K) soil content – also known as NPK sensors – are available as IoT capable solutions^103,104^.

#### Soil pH

The soil pH is a key-indicator in the assimilation of nutrients by plants. The variation in soil acidity is the main factor that determines the availability of nutrients for plants, directly affecting their absorption according to its specific pH range^105^. For example, regarding grapevine crops, the ideal pH range for the best absorption of nutrients by the grapevines is between 5.5 and 6.5^106^.

For real-time measurement of soil pH, low-cost sensors connected to a microcontroller can be used to check the variation in nutrient availability for plants. This provides farmers with a useful tool to timely intervene without the need for laboratory analysis^103,104,107^.

#### Soil electrical conductivity

The use of electrical conductivity sensors are suitable for monitoring the salt content in the soil, due to the conducting properties of this ionic compound^108^. Salt content is a very relevant indicator, as it strongly relates to osmotic potential. More specifically, when salt levels increase due to a combination of irrigation and evapotranspiration, it indicates a decrease in osmotic potential (its value becomes more negative). As a result, the plant is unable to consume water in the desirable quantities, with impacts in the soil nutrients absorption, as well^109^. Conversely, the increase in soil salinization is associated to unbalanced fertilization practices, particularly when nitrogen and potassium chloride-based products are indiscriminately applied^110^.

#### Soil oxygen content

Plants oxygen is affected by either excess or scarcity of water. In turn, the lack of oxygen in the soil can damage roots and make them unable to function normally, leading to slow paced plant growth^111^, with similar effects in the development of grapevines^112^. Under an agronomic perspective, the oxygen level has an influence in the following soil-related aspects: (i) organic matter degradation^113,114^; and (ii) carbon integration^114^. Organic matter is degraded in both aerobic and anaerobic conditions but the presence of oxygen alters the efficiency and dynamics of that process. In an oxygen-free environment degradation becomes more difficult, especially because the organic matter increases its own resistance to decomposition^115^. The lack of oxygen, in turn, also has implications for plants’ nutrients absorption, being considered a limiting factor for the regular phenology cycle^116^. Additionally, plants absorb nitrogen in both ammoniacal ($\hbox {NH}_4^+$) and nitrate ($\hbox {NO}_3^-$) form^117^. However, the oxygen level in the soil affects the nitrogen cycle^118^. Ammoniacal nitrogen ($\hbox {NH}_4^+$) undergoes a bacteria-based nitrification process, involving two oxidization steps, first to nitrites ($\hbox {NO}_2^-$), and then to nitrate ($\hbox {NO}_3^-$). When nitrogen reaches the nitrate ($\hbox {NO}_2^-$) form, it undergoes a denitrification process by facultative anaerobic bacteria, which reduces nitrates ($\hbox {NO}_3^-$) to nitrites ($\hbox {NO}_2^-$) and then to ammonium ($\hbox {NH}_4^+$)^118,119^. Additionally, if the surrounding medium is deficient in oxygen when nitrate ($\hbox {NO}_2^-$) form is shaped up, it can be released into the atmosphere in the form of $\hbox {N}_2$ and $\hbox {N}_2$O by undergoing denitrification by the action of *Pseudomonas* and *Bacillus*^118^. Abstracting this principles and concepts, there are specific sensors capable of measuring soil oxygen^120,121^.

#### Soil temperature

The soil temperature is another important factor in agriculture influencing plants’ properties. In terms of biophysical dynamics, the development of plant roots is affected by low temperatures^122^ and the water available in the soil for the plant relies on temperature variations^123,124^. The temperature of the soil, in turn, influences underground microbiological activity by stimulating or inhibiting the development of microorganisms responsible for the degradation of organic matter^125^ and nitrogen mineralization^126^. It also affects soil respiration by stimulating the development of living and metabolically active underground organisms, responsible for the absorption of $\mathrm {O_2}$ and/or the production of $\mathrm {CO_2}$^127–129^. Another aspect that is influenced by temperature is the stability of soil aggregates^130^ and the availability of nutrients for plants^80,131,132^.

#### Comparative analysis of smart fertilization methods: advantages and disadvantages

In Table 3, the main advantages and disadvantages of each analyzed method are presented. Therefore, sensors for smart fertilization purposes can be selected considering their limitations and strengths.

**Table 3:** Advantages and disadvantages of smart fertilization-related methods.

Method	Advantages	Disadvantages	References
Nutrient content (Electrochemical Sensor)	Rapid response Retrieves a direct measurement of the source component under analysis.	Requires complex laboratory analysis.	^133^
Nutrient content (Optical Sensor)	Non-destructive technique Provides a rapid evaluation in the visible and NIR bands.	The combination of different soil types can negatively influence the readings.	^133^
Nutrient content and soil pH (Soil Electrical Conductivity Measurement)	Rapid response Low-cost solutions are available Resilient sensor in terms of durability.	Reliability varies in different depths Influenced by soil moisture and temperature fluctuations.	^133^
Soil electrical conductivity	No special maintenance is requires With a proper installation, it can be accurate Wide operating and measurement range Typically combines multiple measurements into a single device At the same location, continuous measurements can be ensured.	Temperature variations in wet conditions require constant measurement The inclusion of a data logger may significantly inflate its cost.	^134,135^
Soil temperature	Easy to use Continuous measurements Sensor typically combines multiple measurements into a single device Low latency during operation.	The inclusion of a data logger may significantly inflate its cost.	^77^

### Detection of pests and diseases

Pests and diseases impact in crops production both in terms of quality and quantity, with nefarious effects for agricultural in general, as well as for specific species of high commercial value, such as vineyards^136–138^. Moreover, severe pest and disease attacks can limit the normal development of (but not confined to) the grapevine, reducing its ability to create and store biomass and weakening it progressively^139^. Prevention stems from regular monitoring of weather phenomena and the strategic placement of cameras and microphones in the field allows the farmer to perform interventions in an informed and timely manner^140–143^.

#### Climate-based data monitoring

Climatic conditions are among the factors that most influence the pests and diseases dynamics, in both agriculture and viticulture contexts, and, as such, analyzing climatic parameters is essential to keep the farmer informed about the favorable conditions for the development of certain threats. Currently, there are several models to prevent pests and diseases in the vineyard^144^, including climate-based approaches, which can be used together with IoT systems^140^. The most common models used in IoT systems address downy mildew, powdery mildew, black rot and botrytis^140^, as well as European grapevine moth (*Lobesia botrana*)^145^. Monitoring climatic conditions can also aid in the prediction of the development of auxiliary insects^146^—i.e., known vector agents responsible for the dissemination of infections targeting certain agriculture/viticulture-related crops.

#### Imagery-based detection

IoT-capable sensors for acquiring static or multiframe digital imagery—i.e., cameras—can be used for the detection of potential crop threatening occurrences. In many agricultural contexts, including grapevine crops, such visual data may provide vital capabilities to foil atypical conditions found, for example, in the leaves, caused by pests, diseases, besides other harmful situations like water stress, as previously referred. As such, one or more cameras placed in base supports installed in the field, eventually, considering a confined range of rotation freedom—through an electric motion-capable equipment (e.g. stepper motor or DC motor combined with an encoder) or even a manual adjustment knob—can be applied, depending on the covering area needs^147^.

Among the camera types that can be used for the detection of a wide variety of pests and diseases, more specifically in vineyards, are the multispectral and hyperspectral imagery sensors, as extensively reviewed in^148^. The main focus of their application has been the following threats:

- Multispectral images: grapevine leafroll disease, “flavescence dorée” leafhopper, and armillaria;
- Hyperspectral images: downy mildew, powdery mildew, esca complex, grapevine vein-clearing virus, and grapevine leafroll disease.

Due to the specificities of some diseases, it is necessary to consider specific guidelines for more effective detection. This is the case of vineyard’ downy mildew, in which the “3-10” rule is commonly used to detect the primary infection. Such rule consists in that, when a few conditions are verified, or detected, being one of them the height of the shoot, which should exceed 10 cm. The other conditions are associated with temperature and precipitation. In that regard, Mendes et al.^141^ proposed an approach to estimate cameras acquisition distance through the use of image processing tools capable of determining whether the shoots meet the minimum height required by the “3-10 rule”.

Another use of camera technology combined with image processing and computer vision aims at the detection and quantification of some of the most common pests in the vineyard, such as the European grapevine moth^141,142,149^, green leafhopper, and “flavescence dorée” leafhopper^142^.

#### Sound acquiring devices (microphones)

IoT systems incorporating microphones are used to perform sound-sensitive identification of animals harmful for crops, such as birds, wild boars, deer, among others. Applicable to the generality of crops and, in particular, to grapevines, this type of systems typically use machine learning models trained with the sounds of the animals of interest, to screen and identify the source of the inputs for the microphones. At the same time, if these IoT systems are equipped with sound speakers (preferably waterproof), it is possible to reproduce dissuasive noises—from, for example, eagles, dogs, gunshots, etc.,—to scare away the threatening species. Other identical systems in terms of output are employed to periodically reproduce sounds that are repelling for some threatening animals. However, two main disadvantages are associated to this latter approach: (i) it is unpleasant for those who live or work nearby the sound sources; (ii) the animals learn to get used to the emitted sounds after a while, leading to the loss of purpose^143^.

#### Comparative analysis of pests and diseases detection methods: advantages and disadvantages

A comparative analysis regarding the methods used in the detection of pests and diseases is presented in Table 4, in which the main advantages and disadvantages of each one is highlighted. Therefore, having considered this set of benefits and limitations, professionals/practitioners will be able to make informed decisions regarding the selection of this kind of sensors and the respective goal-oriented implementation.

**Table 4:** Advantages and disadvantages of pests and diseases detection methods.

Method	Advantages	Disadvantages	References
Climate-based data monitoring	Significantly optimizes the use of pesticides Enables preventive actions before outbreaks Can be used to monitor large areas simultaneously Increases profitability and management efficiency.	Depends on accurate meteorological and historical data Requires specialized technical knowledge Accuracy varies according to region/topography A significant investment is required for implementation and training.	^150^
Imagery-based detection	Provides a higher resolution Easy to install There are low-cost solutions available (covering, for example, RGB and multispectral sensors) Allows flexibility for specific pests detection training A single device can detect more than one type of occurrence (multi-functional).	Limited coverage compared to mobile platforms capable of transporting such kind of data acquisition devices (e.g. unmanned aerial vehicles – UAV) Cameras require calibration to a correct acquisition angle Several environmental conditions (poor illumination, fogginess, dirt-inducing dust particles, etc.) can significantly limit the use of cameras.	^151,152^
Sound acquiring devices (microphones)	Allows flexibility for specific pests detection training A single device can detect more than one type of occurrence (multi-functional)	For an effective operation, multiple microphones/sound acquisition systems must be spread across the croplands (e.g. vineyards).	^143,153^

### Vegetation cover mapping and monitoring

Vegetation indices (VIs) are valuable data obtained by analyzing specific wavelength bands belonging to the visible or invisible ranges of the electromagnetic (EM) spectrum that allow farmers to highlight information about the vegetation cover status^154^. Their utility is immense and diversified^155^. In agronomy, the wavelength bands with the greatest applicability in the visible part of the EM spectrum (RGB), ranging from 450 to 750 nm, are the following: blue (450–495 nm), green (495–570 nm) and red (620–750 nm). Measured through RGB cameras, VIs operating in these wavelengths can be used to determine chlorophyll concentration, which allows monitoring the health of the leaves and, consequently, of the plant^156^. RGB images can also be used to estimate leaf area and to correlate the plant water status through the reduction of the Dark Green color space [R126 G134 B68; R116 G123 B75], which has a higher correlation with stem water potential, also observable by the leaf angle changing as a response to water stress^157^.

Outside of the visible range, the near and medium infrared wavelength band (850–1700 nm) have been of great relevance^158^. In the context of precision agriculture, NIR (Near-Infrared) plays a crucial role in assessing plant health and vigor. Vigorous plants, in full development and with intense photosynthetic activity, generally exhibit higher levels of vegetation reflectance in the NIR range^159^. This property, when analyzed together with data obtained in the visible spectrum (RGB), allows for the calculation of various vegetation indices (VI). These indices are based on the differences in vegetation reflectance at various wavelengths, providing valuable information about the state of crops^160,161^. For example, the Normalized Difference Vegetation Index (NDVI), according to Eq. 3, considers, near-infrared (NIR) – besides red-related bands.

3 $$\begin{aligned} NDVI = \frac{NIR-Red}{NIR+Red} \end{aligned}$$

NDVI can diagnose the state of conservation/degradation of the vegetation cover, allowing the determination of the density of photosynthetically active plant material^162^. In the scope of viticulture, during the final stages of the grapevines vegetative cycle, Normalized Difference Red Edge index (NDRE) should be preferred over NDVI, as it better captures the vigor and chlorophyll content of plants in the final stages of development^163^. It combines NIR and red-edge (RE), as shown in Eq. 4.

4 $$\begin{aligned} NDRE = \frac{NIR-RE}{NIR+RE} \end{aligned}$$

Such NDVI and/or NDRE strategy alteration is recommended because the higher and more vigorous the vegetation is—also measurable through another VIs like Leaf Area Index or LAI—, the greater are the limitations of NDVI and, conversely, the better is the response of NDRE^163^. NDVI can also be used together with estimated soil electrical conductivity, to identify spatial variability within a plot, towards the proposal of prescription maps for time-effective and location-aware interventions, capable of providing guidelines for personalized irrigation or fertilization^164^. Still regarding the inclusion of non-visible parts of the EM to process vegetation imagery, another work proposed by Wong et al.^165^ resorts to multispectral cameras installed on a tower, to determine the behavior of the Photochemical Reflectance Index (PRI) in response to water stress and post-water stress recovery in grapevines. This way, PRI can complement irrigation management methods based on indicators such as CWSI and evapotranspiration.

Vegetation cover mapping and monitoring consists of a set of spectral/spatial analysis methods. Based in the literature^166–174^, several advantages can be highlighted in that regard, as for example:

- Suitability for extracting of long-term series of consistent and comparable data;
- Sensitivity to the detection of changes in vegetation cover;
- Adequacy to evaluate agricultural productivity;
- Adequacy to perform crops health monitoring;
- Proneness to detect leaks in irrigation systems, when combined with other data sources;
- Support of a variety of tools available to assess several vegetation aspects, with distinct applications in viticulture and agriculture.

Although, many other disadvantages should be considered when resorting to spectral/spatial analysis for performing vegetation cover mapping and monitoring. The following list underlines some of them:

- Limitations in the ability to distinguish biomass levels in dense vegetation (saturation points);
- Proneness to be affected by atmospheric particles, such as dust and water vapor (atmospheric interference), which often impact on data quality;
- Susceptibility to the presence of water in vegetation and soil, which may negatively influence spectral reflection and, therefore, the use of VIs;
- Susceptibility to soil morphology, which may affect the reflection responses and, therefore, the use of VIs;
- Proneness to spectral responses variations induced by the frequent presence of organic matter in the soil;
- Highly dependent on spatial and spectral resolution;
- Some high-resolution sensors, such as hyperspectral cameras, are still costly.

### Climatic indices monitoring

The ability to monitor climate conditions is of utmost importance for predicting water use, gathering relevant data for common decision support system (DSS), and understanding conditions that might lead to the onset of common pests and diseases. Considering the due location awareness, interventions accuracy can be optimized in several agricultural crops, and more specifically, in viticulture^175^, through IoT sensors capable of collecting data for supporting winemakers’ decisions.

The maturation of grapes—and, consequently, their quality—is extremely influenced by the climate variations occurring from location to location, particularly in terms of air temperature and relative humidity, and solar radiation, both at the microclimate and at the mesoclimate levels^176^, with the content of anthocyanins and the Brix level being the most affected parameters^177^. While bioclimatic indices are useful for representing the risks and potentialities of climate effects in a given region, microclimatic indices are extremely useful for representing the dynamics of the climate at the level of each plant individually^176^.

Given the current challenges associated with climatic changes, it is increasingly necessary to adopt monitoring, characterization, and categorization approaches to better assess how these factors will interfere in the adaptation and development of plants in specific locations and, consequently, anticipate the potential of certain geographies for the cultivation of grapevine in the coming years^178^.

While the Tonietto and Carbonneau^179^ deepens the bioclimatic indices, Matese et al.^176^ discusses the microclimatic indices in detail. Both topics can be found briefly summarized as follows:

- Night cold index: it can be used to characterize the qualitative potential of the location where the vineyard is located, by relating the average of minimum temperatures—during the month in which grape maturation normally occurs—with the formation of aromatic and polyphenolic compounds. The results obtained from this index can be compared resorting to classes of intervals, where the effect of a given climate on the potential for loss or gain of aromas and polyphenols in white and red wines is observable if the heliothermal conditions are adequate for grape maturation^179^.
- Huglin heliothermal index: it is used to relate the location to vineyards potential for sugar production and acidity levels decreasing during grapes development, making it a good indicator for maturation status. More specifically, when used in conjunction with the *night cold index*, it allows to discriminate the effect of the climate on the potential evolution of sugars, acidity, aromas, and polyphenols that occur during grape maturation^179^.
- Dryness index: specifically created for grapevine cultivation, this index establishes an association between the location and vineyards’ potential capacity to store water in the soil, within a given period. Despite of having an application that is not directly related with such water storage assessment, this index takes into account important factors in irrigation management, such as rainfall levels, water transpiration by plants, soil water evaporation, and initial soil water reserves^179^. This way, it is possible to characterize a region in terms of its water availability level, as well as monitor its temporal evolution^178^, considering the specific needs of the grapevine and its sensitivity to hydric variations in soil.
- Hydrothermic index of Branas, Bernon, Levadoux: developed for the characterization of an area in terms of production quality and quantity, this index relies in the interaction of temperature and precipitation, during the most critical period of grape production, i.e. between April and August^180^. Moreover, it is also used to assess the risk of occurrence and proliferation of downy mildew, which is one of the most common and destructive diseases affecting grapevines^180,181^.
- Microclimatic indices: these indices are addressed in^176^ and relate the location to grapes potential quality of individual grapevine plants, with the advantage of considering comparisons regarding the effects of vineyard operations on the resulting fruit. To that end, temperature sensors are placed in contact with the grape cluster – but not inside the fruit—at the initial stages of formation. Additionally, another similar sensor is placed near of the vegetative wall, for monitoring the environmental temperature. Finally, to infer data regarding a grape as if it was exposed, a radiometric sensor operating in the spectral range of 400-1100 nm is also positioned nearby a grape cluster. The most proper microclimatic indices in terms of representativeness and potential to discriminate effects of vineyard practices are the following:
  - CR dBDi (Dynamic Cluster Radiation Broad Daylight Index): it allows to analyze the average solar radiation intercepted by a grape clusters around solar noon. This index is highlighted as the one that best explains the microclimatic effects;
  - CT dBDi (Dynamic Cluster Temperature Broad Daylight Index): it enables the analysis of the average temperature of a grape cluster around solar noon. This index also showed a satisfactory precision, even though the results are not so robust and statistically significant as the ones presented by CR dBDi;
  - CR Range (Cluster Radiation Range): it is used to measure the exposure of a cluster to solar radiation throughout the day.

Processes and calculations can be found duly detailed in the respective provided literature references^176,179^.

It is worth highlighting that some off-the-shelf sensors enabling cost-effective climate monitoring and compatible with IoT integration are available^33^.

The climatic indices monitoring, essentially, consists of a weather analysis methods group. According to the literature^182–185^, the following advantages can be highlighted for such group:

- Great capacity for anticipating critical issues months prior to their manifestation, even before the crops growing season;
- Proneness to provide insights of the influence of local climate on grape and wine quality;
- Adequacy to aid in the outlining of viticultural strategies that can be applied to settle a vineyard (e.g., altitude, orientation, rootstock, grape varieties, etc.).

As for the disadvantages, a main limitation is noteworthy, which is related with the need of accurate data for the sake of climatic models effectiveness. Additionally, when remote or understudied areas are involved, achieving trustworthy data can be quite challenging.

The following section will present relevant guidelines for sensors selection, according to PA/PV applications, techniques and parameters/variables to monitor.

## PA/PV applications, techniques, and measurements: a quick guide towards the selection of IoT-capable sensors

Within the scope of PA/PV, various sensor-based methodologies can be considered for addressing a wide range of challenges, in some cases, with overlapping applications, as it can be inferred from Fig. 1. In practical scenarios, this diversity can lead to uncertainties and indecision when it comes to the selection and configuration of the most appropriate solution. Therefore, Tables  5, 6 and 7 congregate a handy set of information to facilitate the connection between IoT-capable sensors and applications, implementation methods, and associated variables.

Starting from Table 5, to implement smart irrigation, one must consider that different techniques will drive to the measurement of different parameters. For example, when resorting to crop evapotranspiration technique, the following parameters must be considered: air temperature and relative humidity, wind speed, and solar radiation. Regarding atmospheric pressure, it is not of mandatory use for determining crop evapotranspiration, but its inclusion can lead to more accurate results. This whole combination of techniques and sensor-based parameters aims to provide data towards the optimization of water use while minimizing waste.

**Table 5:** Smart irrigation application, its techniques and types of IoT sensors needed for each case.

App	Tech	SWCS	SWTS	ATS	ARHS	WSaDS	SRS	APS	SFS	LTS	MTS	LTuS	D	WLS	WTS	References
Smart irrigation	Soil water content	$\checkmark$	–	–	–	–	–	–	–	–	–	–	–	–	–	^23,26,27,103,104^^73,108,109,120,121^
	Water tension in the Soil	–	$\checkmark$	–	–	–	–	–	–	–	–	–	–	–	$\oplus$	^26,30,73^
	Crop evapotranspiration	–	–	$\checkmark$	$\checkmark$	$\checkmark$	$\checkmark$	$\oplus$	–	–	–	–	–	–	–	^32,33,186^
	Sap Flow	–	–	–	–	–	–	–	$\checkmark$	–	–	–	–	–	–	^45,187,188^
	CWSI	–	–	$\checkmark$	–	–	–	–	–	$\checkmark$	–	–	–	–	–	^47,189^
	Stem Water Potential	–	–	–	–	–	–	–	–	–	$\checkmark$	–	–	–	–	^60^
	Leaf Turgor	–	–	–	–	–	–	–	–	–	–	$\checkmark$	–	–	–	^63^
	Dendrometry	–	–	–	–	–	–	–	–	–	–	–	$\checkmark$	–	–	^24,67^
	Water level in reservoirs	–	–	–	–	–	–	–	–	–	–	–	–	$\checkmark$	–	^73^

Cells symbols: $\checkmark$, mandatory parameter; and $\oplus$, optional parameter.

Header acronyms: App, application; Tech, technique; SWCS, Soil water content sensor; SWTS, Soil Water Tension Sensor; ATS, Air Temperature Sensor; ARHS, Air Relative Humidity Sensor; WSaDS, Wind Speed and Direction Sensor; SRS, Solar Radiation Sensor; APS, Atmospheric Pressure Sensor; SFS, Sap Flow Sensor; LTS, Leaf Temperature Sensor; MTS, Microtensiometer Sensor; LTuS, Leaf Turgor Sensor; D, Dendrometer; WLS, Water Level Sensor; WTS, Waterproof Temperature Sensor

Table 6 outlines the relevant methods and variables to consider for maximizing plant nutrition efficiency, in the perspective of smart fertilization. For example, nutrient content can be determined using hyperspectral cameras and NPK sensors.

**Table 6:** Smart fertilization, associated techniques and types of IoT sensors advisable for each case.

App	Tech	NPK	pH	SECS	WTS	STS	HSC	References
Smart Fertilization	Nutrient Content	$\checkmark$*	–	–	–	–	$\checkmark$**	^100,101,103,104^
	Soil pH	–	–	$\checkmark$	–	–	–	^103,104,107^
	Soil electrical conductivity	–	–	–	$\checkmark$	–	–	^73,103,104,108,109^
	Soil Oxygen Content	–	–	–	–	–	$\checkmark$	^120,121^
	Soil Temperature	–	–	–	–	$\checkmark$	–	^73,103,104,108,109^

Cells symbols: $\checkmark$, Advisable sensor; $\checkmark$*, Includes the capacity for quantifying N,P and K macronutrients; and $\checkmark$**, Includes the capacity for determining most of the macro and micronutrients of interest.

Header acronyms: App, application; Tech, technique; HSC, Hyperspectral Camera; NPK, NPK Sensor; pH, pH sensor; SECS, Soil Electrical Conductivity Sensor; WTS, Waterproof Temperature Sensor; and STS, Soil Temperature Sensor

Table 7 summarizes the data-based methods with importance for decision-making and strategy development aimed at more efficient and sustainable agricultural practices concerning to: (a) pests and diseases detection; (b) monitoring and mapping of vegetation cover; and (c) climatic indices monitoring. For example, to perform the detection of pests and diseases, climate monitoring approaches, cameras and microphones can be used. Within cameras option, RGB sensors are adequate to detect visible manifestations of threats, while hyper/multispectral devices are usually required for inspections out of the visible EM spectrum range.

**Table 7:** Detection of pests and diseases, vegetation cover monitoring and mapping and climate indices monitoring applications, their techniques and type of IoT-capable sensors advisable for each case.

App	Tech	SWCS	ATS	ARHS	WSaDS	SRS	P	RGBC	MSC	HSC	Mic	WTS	References
DPD	Climate Monitoring	–	$\checkmark$	$\checkmark$	$\checkmark$	$\checkmark$	$\checkmark$	–	–	–	–	–	^33,140,146^
	Cameras	–	–	–	–	–	–	$\checkmark$*	$\checkmark$**	$\checkmark$**	–	–	^141,142^
	Microphones	–	–	–	–	–	–	–	–	–	$\checkmark$	–	^143^
VC	Cameras	–	–	–	–	–	–	$\checkmark$*	$\checkmark$**	$\checkmark$**	–	–	^157,163,165^
CIM	Bioclimatic Indices	$\checkmark$	$\checkmark$	$\checkmark$	$\checkmark$	$\checkmark$	$\checkmark$	–	–	–	–	–	^33,175,177^
	Microclimatic Indices	–	$\checkmark$	–	–	$\checkmark$	–	–	–	–	–	$\checkmark$	^176^

Cells symbols: $\checkmark$ - Advisable sensor; $\checkmark$* - Sensors for visible manifestations; and $\checkmark$** - Continuous narrow-band and discrete wide-band sensors operating in ranges that typically extend beyond the EM’s visible interval to inspect spectral occurrences that cannot be detected at naked eye.

Header acronyms: App, application; Tech, technique; SWCS, Soil water content sensor; ATS, Air Temperature Sensor; ARHS, Air Relative Humidity Sensor; WSaDS, Wind Speed and Direction Sensor; SRS, Solar Radiation Sensor; P, Pluviometer; RGBC , RGB Camera; MSC, Multispectral Camera; HSC, Hyperspectral Camera; Mic, Microphone; and WTS, Waterproof Temperature Sensor.

Target tasks (bellow the “App” row of the header): DPD, Detection of pests and diseases; VC, Vegetation Cover Monitoring and Mapping; and CIM, Climate Indices Monitoring;

After identifying the required parameters for the diverse monitoring applications and techniques oriented to gather critical data for the characterization of agricultural/viticultural contexts, the specific off-the-shelf sensors allowing to attain such for measurements need to be addressed towards an effective guidance of practitioners, professionals and researchers in the selection of the proper devices set regarding specific intentions/purposes. Therefore, Tables 8,  9 and  10 link concrete available IoT-capable sensor models and type to the specific variables to measure. Dividing such information into the three designated tables aims to manage the extensive information effectively, facilitating easier visualization and interpretation by those who might be interested in developing PA/VP IoT-based solutions.

**Table 8:** IoT sensor types and corresponding examples of available devices identified in the surveyed literature(part 1/3).

Soil water content sensor	Soil water tension sensor	Air temperature sensor	Air relative humidity sensor	Wind speed and direction sensor	Solar radiation sensor	Atmospheric pressure sensor	Pluviometer
Acclima Digital True, TDR-315H^121^	Decagon, MPS-1^31^	Bosch, BME280^142^	Bosch, BME280^142^	Vaisala, Ultrasonic Wind Sensor Model WMT700^186^	Apogee Instruments, Apogee SP-110^7^	Bosch, BME280^142^	Sparkfun, Weather meters^33,190^
Campbell Scientific, CS610^120^	Decagon, MPS-2^73^	Kuong- shun Electronic, DHT11^190^	Seeds- tudio, DHT22^33^	Sparkfun, Weather meters^33,190^	Perkin- Elmer Optoelectronics, VTP4085^176^	Bosch, BMP085^33^	-
Decagon, 5TE^108,109^	Irrometer, Watermark 200SS^30^	Seed- studio, DHT22^33^	Vaisala, HMP155^186^	–	–	Vaisala, PTB330^186^	–
Decagon, 10HS^73^	–	Vaisala, HMP155^186^	–	–	–	–	–
Decagon, EC-5^95,109^	–	Vaisala, HUMITER 50Y^157^	–	–	–	–	–
Decagon, ECH2O-5TE^109^	–	Unknow, Type T thermocouple^176^	–	–	–	–	–
Decagon, GS3^73,109^	–	–	–	–	–	–	–
JXCT, soil integrator sensor^103,104^	–	–	–	–	–	–	–
Sentek, Drill & Drop^175^	–	–	–	–	–	–	–
Stevens- Water, HP II^73^	–	–	–	–	–	–	–

In each cell, straightforward information regarding sensor provider (manufacturer or seller), type and works in which they were used can be found.

**Table 9:** IoT sensor types and corresponding examples of available devices identified in the surveyed literature(part 2/3).

Leaf wetness sensor	Sap flow sensor	Leaf temperature sensor	Microtensiometer	Leaf turgor sensor	Dendrometer	Water level sensor	RGB camera	Multispectral camera
Campbell Scientific, 237-L^191^	Dynamax, sap flow sensor^45^	FLIR, A300 Infrared Camera^189^	FloraPulse^60^	Yara, ZIM Probe^64^	Ecomatik, DD-L^24^	Sensotec Instruments, LMK^73^	Basler Vision Technologies, scA1600-14gc^157^	Holland Scientific, Crop Circle ACS-430^163^
Haiwang, FC-37^190^	Greenspan, SF200^187^	Apogee, IRT-P5^189^	–	–	–	–	Shenzhen Technology, ELP 2.2MP USB Camera 2.8 mm focal length^141^	Ocean Optics, Flame VIS-NIR Spectrometer^165^
–	ICT International, SFM1^188^	Bio Instruments, LT-1M^189^	–	–	–	–	Shenzhen Technology, ELP 2.2MP USB Camera 3.6 mm focal length^141^	Specim., Spectral Camera V10^100^
–	Dynamax, Thermal Dissipation Probe^24^	Apogee, SI-121^7^	–	–	–	–	Huiber Vision Technology, HVBCAM 5.0MP USB Camera with a 160-degree fish-eye lens^141^	Vossk$\ddot{u}$hler GmbH, NIR-300PGE^157^
–	–	Sony, IMX219^55^	–	–	–	–	–	–
–	–	Flir, A35 Infrared Camera^54^	–	–	–	–	–	–

In each cell, straightforward information regarding sensor provider (manufacturer or seller), type and works in which they were used can be found.

**Table 10:** IoT sensor types and corresponding examples of available devices identified in the surveyed literature(part 3/3).

Hyperspectral camera	Microphone	NPK sensor	Soil pH sensor	Soil electrical conductivity sensor	Temperature sensor (water proof)	Soil oxygen sensor
BaySpec, OCI Imager (OCI-UAV-D1000)^101^	Waterproof microphone (not specified,^143^	JXCT, soil integrator sensor^103,104^	JXCT, soil integrator sensor^103,104^	Decagon, 5TE^108,109^	Decagon, 5TE^108,109^	Apogee, SO-411 O2^121^
Specim., OLE-23 VNIR Enhanced Series^100^	–	–	–	Decagon, ECH2O-5TE^109^	Decagon, ECH2O-5TE^109^	Environ- mental Measurement Japan, MIJ-03^120^
–	–	–	–	Decagon, GS3^73,109^	Decagon, GS3^73,109^	–
–	–	–	–	JXCT, soil integrator sensor^103,104^	JXCT, soil integrator sensor^103,104^	–
–	–	–	–	Steven- sWater, HP II^73^	Stevens- Water, HP II^73^	–

In each cell, straightforward information regarding sensor provider (manufacturer or seller), type and works in which they were used can be found.

Considering the revised works, one can infer that there are several options associated to soil water content, air and leaf temperature sensors. A wide range of equipment is also available for measuring soil electrical conductivity and waterproof temperature sensors. RGB and multispectral sensors can also be provided by a large range of companies operating in this market. Less abundant are the options for pluviometers, dendrometers, microphones, and sensors related to water level, NPK and soil pH sensors. This scarcity may be caused by either technological advancements or the reduced interest in these types of sensing approaches by the community orbiting around PA/PV-oriented IoT technologies. The following section will address the recommended procedures and cares to be taken in the installation of these sensors, when setting up an IoT (or IoT-like) system for PA/PV environment monitoring.

## IoT-capable sensors installation: a practical guide

Correct installation of IoT-capable sensors is an important step to ensure the efficacy of monitoring systems, playing a fundamental role for the adequate and consistent data gathering underlying decision-making support, towards agriculture/viticulture sustainable practices. Strategic positioning of sensors should be aligned with the characteristics of the location where it is installed, ensuring precision and reproducibility, while minimizing interference and unnecessary costs^192^. Therefore, this section is dedicated to the discussion of recommended procedures and cares to be taken while installing PA/PV sensors, namely, focusing the ones addressed in the previous section of this study.

### Soil water content and soil matric potential sensors

The ideal placement of soil moisture sensors should consider the soil water retention characteristics representative of the entire terrain, which can be assessed through soil organic matter content and texture. Under conditions wherein only a single location can be monitored, it is recommended to search for the one with the least capacity for water retention^193^.

**Fig. 2:**
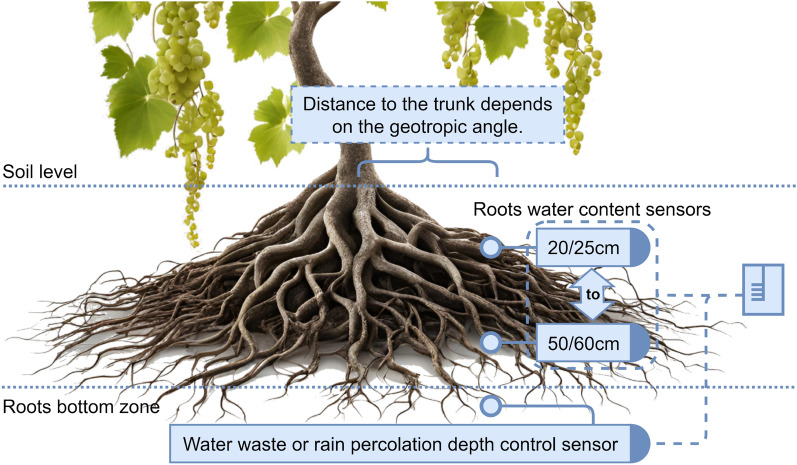
General guidelines for the installation of soil water content and soil matric potential sensors. Note: the incorporated vine plant representation was generated with the support of the *Canva* platform for the main design (“Home - Canva”, accessed in July 2024. Available at https://www.canva.com/), and the *Craiyon* platform for background removal (“Free Background Remover - Craiyon”, accessed in July 2024. Available at https://www.craiyon.com/).

The soil water content and soil matric potential can be analyzed at various depths, depending on the measurement objective^194^. The sensors should be placed nearby plants’ roots, which, in the case of the grapevine, is roughly 50–60 cm bellow soil surface, and considering a very low root density in the first 20–25 cm. Furthermore, to determine the horizontal distance of the installation to the trunk, the position of the roots should be assessed considering the plant geotropic angle, which, in turn, is influenced by the specific type of applied rootstock^21^. Such guidelines can be found graphically depicted in Fig. 2.

It may also be advantageous to place a depth sensor at the bottom of the root zone, which will serve as an indicator of the amount of water that reaches the base of the roots, whether by irrigation or precipitation. Obtaining such data is useful for showing possible water waste or measuring the percolation depth of water from rain, taking into account the effects on soil leaching, as it transports nutrients outside the root zone^193^.

For an increased accuracy on soil water content measurements, it is essential to look for areas with the least possible soil disturbances, as such occurrences require time to consolidate the IoT-capable sensor installation. In that regard, soil settling can be fostered through some irrigation or heavy rainfall^195^. It is worthy to highlight that this stage requires careful planning and procedures. To install any sensor beneath the soil surface, it is necessary to open a hole at a desired depth, which inevitably modifies the bulk density and texture of that land portion, which may take weeks or months to recover until attaining its default compaction. This difference affects the effectiveness of the sensor in measuring the soil water content^196^.

### Air temperature and relative humidity sensor

The air temperature and humidity sensor should be placed inside a ventilated capsule that, at the same time, provides protection from solar radiation. The sensor should be installed at 2 m high, and at a distance equivalent to, at least, 4 times the height of potentially obstructive nearby elements. Moreover, in the presence of structures or entities capable of influencing air temperature and humidity—such as some kind of pavements and water zones—, a minimum distance of 30 m from the sensor to these spots should be kept^194^.

### Wind speed and direction sensor

The wind sensor should be installed at 2 m high, at a minimum distance equal to 10 times the height of potential obstructions^194^.

### Solar radiation sensor

The solar radiation sensor should be installed at less than 3 m high, as far as possible from objects producing shadows towards the device, considering, also, that the solar angle changes throughout the year. In addition to mitigating shadows of nearby objects (if any), the sensor should also be kept away from reflective surfaces or artificial radiation sources. In the Northern Hemisphere, the sensor should be installed on the south side of the main station, to avoid its shading^194^.

### Atmospheric pressure sensor

The atmospheric pressure sensor should be installed at 1 m high, away from any type of obstruction^186^.

### Sap flow sensor

The installation of a sap flow sensor should be carried out at night, when the sap flow is lower, in order to prevent embolisms^197^. Before installing the sensors, it is necessary to determine the depth of the xylem vessels in the trunk. During installation, it is necessary to remove the bark residuals from the trunk, ensure that the holes where the sensors will be installed are aligned parallel to each other and as accurately as possible. Additionally, the length between each hole needs to be as precise as possible. The sensors should not be installed in the nodes or in dead wood^188^.

When the installation kit includes hollow aluminum tubes, these should be placed in the trunk, more specifically, in the holes. Then, the sap flow sensor probes should be lubricated with heat-conducting paste and then inserted into the cylindrical tubes previously attached to the trunk^198^. Moreover, the probes or aluminum tubes should be covered with alcohol (minimum 70% of vol.) or a fungicide before insertion into the trunk^197,199^.

When the employed method is the heat pulse one, two temperature measurement sensors are used along with a heat sensor. One of the temperature sensors should be placed 5 mm above the heat sensor, while the other should be installed 10 mm below it^200^.

In the case of the Granier heat balance method, only two trunk-attachable sensors are used—one with heating system incorporated and another without it—placed with a distance of 10 cm from each other^201,202^.

To aid in sensor positioning, to prevent location drifts, and to suppress air entry into the xylem vessels, the sensors should be tied to the the trunk with adhesive tape^197,198^. At the end of the installation, to protect the sensor from environmental influences, radiation shields made of bubble aluminum foil should be used^198^.

It is noteworthy that the location of the sensors should be changed regularly, to mitigate the damage caused by the installation and usage.^202^.

Diving into a concrete example regarding the installation of a sap flow sensor in a vine trunk, Ouadi et al.^203^ reports a series of steps resorting to the EXO-Skin ™device, from Dynamax, Inc. The first step is to select a vine cordon that corresponds to the wood from year $N-2$, with the aim of avoiding irregularities in the basal trunks while simultaneously mitigating the effects of soil temperature gradients. Then, the sensor’s heating sleeve should be carefully wrapped around the vine stem after removing the bark to expose the inner wood. Sleeve’s flexibility ensures a precise fit on each section of the stem, allowing the heat to be distributed uniformly and radially across the inner wood. Prior to applying thermal and waterproof protection, it is crucial to use thermal grease to optimize the contact between the sensor and the wood. Following the sensor accommodation, the trunks, cordons, and stems should be wrapped with an aluminum sheath to minimize direct solar radiance, which can potentially affect the readings.

**Fig. 3:**
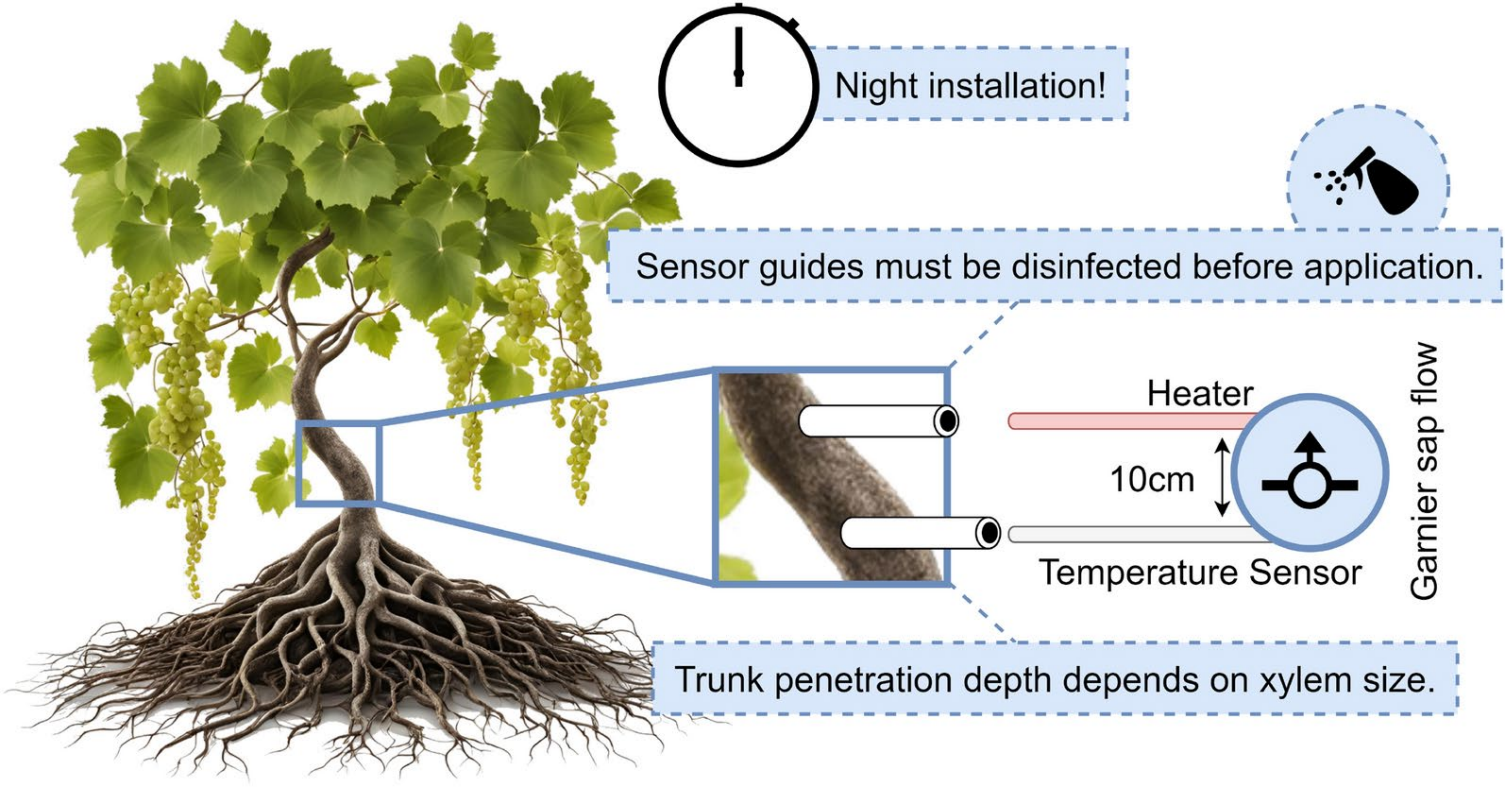
General guidelines for Granier-based sap flow sensor installation. Note: the incorporated vine plant representation was generated with the support of the *Canva* platform for the main design (“Home - Canva”, accessed in July 2024. Available at https://www.canva.com/), and the *Craiyon* platform for background removal (“Free Background Remover - Craiyon”, accessed in July 2024. Available at https://www.craiyon.com/).

Fig. 3 graphically depicts the general guidelines for the installation of a Granier-based sap flow sensor.

### Leaf temperature sensor

Leaves temperature is influenced by the plants’ physiological structure plants and by exogenous factors, such as solar radiation, air temperature, and wind. Therefore, to collect representative data regarding the temperature of leaves, the sensing device should be installed on foliage that is directly exposed to sunlight, ensuring that it is not obstructed by the canopy^189^.

### Leaf wetness sensor

The leaf wetness sensor should be installed at 30 cm high, in a location safeguarded from irrigation systems or other sources of moisture. In the Northern Hemisphere, the sensor should be installed facing north, at a 45°angle relative to the ground^194^.

### Microtensiometers

The installation of a microtensiometer sensor involves several precise steps to ensure accurate readings and minimal damage to the plant. Initially, bark needs to be taken out from a flat section of the trunk, followed by the removal of phloem tissue using a cork borer and a spatula or a blade. A custom stainless steel sleeve, with an outer diameter of 14 mm and an inner diameter of 9 mm, must be then inserted using a hammer. The sleeve serves as a guide for drilling approximately 5 cm into the trunk until the xylem tissue, removing the resulting residues. To ensure good contact with the sensor, the cavity is filled with a kaolin-based mating compound. The hydrated sensor probe is then inserted into this compound. Afterwards, stainless steel cap is placed to close the sleeve, and its exterior is sealed with silicone to prevent air and water exchanges. To minimize temperature fluctuations affecting the sensor, the installation is wrapped using plastic foil, followed by a 25 mm-thick foam batting with reflective aluminum foil. The sensors typically require two days to level up with the vine parameters^60,62^.

### Leaf turgor sensor

To install leaf turgor sensors, it is necessary to follow some specific precautions. First, for a given plant, a healthy and mature leaf needs to be selected from the inner part of the canopy, avoiding the ones directly exposed to sunlight. The sensor is then carefully placed on the selected leaf, ensuring that the wrapping of main veins is avoided. Depending on the type of sensor used, it may be necessary to adjust the initial pressure of the probe (*Pp*) as an initial calibration procedure. For example, in the case of the ZIM-probe, the initial pressure can be adjusted to a value between 10 and 25 kPa. Finally, a wireless data receiver is fixed in an appropriate position on the plant’s trunk, connecting to the sensor and allowing the continuous transmission of collected data to a central server^204^.

### Dendrometer

Dendrometers require calibration before installation, using a precision micrometer or metal plates with known thickness. They should be placed away from the ground to avoid interference from pests that may exist and scars caused by grafting, pruning, etc. The bark tissue that protects the trunk (rhytidome) should be removed in the area where the dendrometer will be installed, to ensure an effective contact with the trunk. It is advisable to place these devices aside of the stem, as much as possible safeguarded from the sun, to minimize the negative effects of direct solar radiation heating. A spring or cyanoacrylate glue should be used to conveniently tie the dendrometer to the trunk. Moreover, the entire area around the dendrometer should be covered with insulating materials and a thermal protector to minimize direct solar radiation heating and avoid the impact of raindrops. Depending on local conditions and the precision required for measurements, it may be advisable to record temperature fluctuations of the support and wood near the dendrometer, to evaluate the thermodynamic effect regarding heat spreading^66^.

### Water level sensor—for reservoirs

Water level sensors should be placed without obstacles nearby and preferably located in the center of the reservoir^205^. In order to ensure that there are no external interference in the sensor reading, the water level can be measured by placing the sensor inside a perforated tube. This operation must ensure that the water level inside the tube is in equilibrium with the water level of the reservoir^79^.

### NPK, pH, and electrical conductivity of soil sensors

Works pointing out good practices for installing NPK, pH, or soil electrical conductivity could not be found. However, there are authors who suggest taking soil samples from known terrain portions for laboratory analysis to determine the three mentioned parameters, and elucidate regarding suitable placement sites for sensors installation^206,207^.

To proper analyze the content of nutrients, pH and/or soil electrical conductivity, soil samples around plants roots should be taken. The number of samples depends on the soil heterogeneity and the desired certainty degree. Precautions should be taken while collecting samples at different stratification levels corresponding to distinct soil or root occupation profiles^206^.

When dealing with grapevines, the root-related substrate is roughly located between 50-60 cm depth, while the first 20-25 cm layer has a very low root density. These considerations, along with the position of the roots through the assessment of the plant geotropic angle, which, in turn, is influenced by the specific type of applied rootstock^21^ are important to determine the horizontal distance of the sensor’s installation to the trunk.

### Soil oxygen sensor

The scientific literature addressing soil oxygen sensors installation guidelines in vineyards seems to be scarce. However, a few guidelines can be inferred supported by previous sensors analysis and a few literature contributions on particular crops. More specifically, works on avocado orchards were considered, due to the resemblance of the characteristics of the implied crops’ roots comparatively to vine plants, namely, in what regards to water penetration and nutrient absorption. Most of these trees have absorbing roots located within the first 60 cm of soil, despite they can extend beyond 1.5 m deep^208^. In^209^, wherein a soil oxygen sensor was installed together with a drip irrigation system in an avocado orchard, the recommend to bury the sensing device around 35 cm deep. Furthermore, to determine the horizontal distance of the installation to the trunk, the position of the roots should be assessed considering the plant geotropic angle, which, in turn, is influenced by the specific type of applied rootstock^21^.

While transposing to grapevine roots, one should acknowledge that most of the nutrients and a significant portion of the available water in the soil is absorbed in depths ranging from 50 to 60 cm below the surface. After that range, the presence of roots capable of absorbing the water necessary for maintaining the various physiological functions of the plants—especially during the summer period when there is less water available in the surface horizons—starts to gradually decrease^21^.

### Soil temperature sensor

The optimal installation depth for soil temperature sensors depends on the specific objective of the measurement. Although soil temperature is commonly measured at a depth of 10 cm, different soil stratification levels may require measurements at other depths, being the root-comprising layers the ones that best represents plant’s conditions. In terms of installation procedure, the ideal location for placing the soil temperature sensor should be a flat surface of 1 $\hbox {m}^2$, in the center of a circular area with a radius of 10 m. It is also important to consider that the installation of the sensor should be done, at least, 1.5 m away from edified structures (e.g. towers) and as far as possible from natural runoff zones^194^.

### Rain gauge sensor—pluviometer

The rain gauge should be installed at a height of 1 m, and at a minimum distance equal to 4 times the height of the nearest potentially obstructive element. It should be leveled in relation to the ground plane to maximize exposure to the sky. Any additional securing or protective equipment must keep the top of the device clear^194^.

**Fig. 4:**
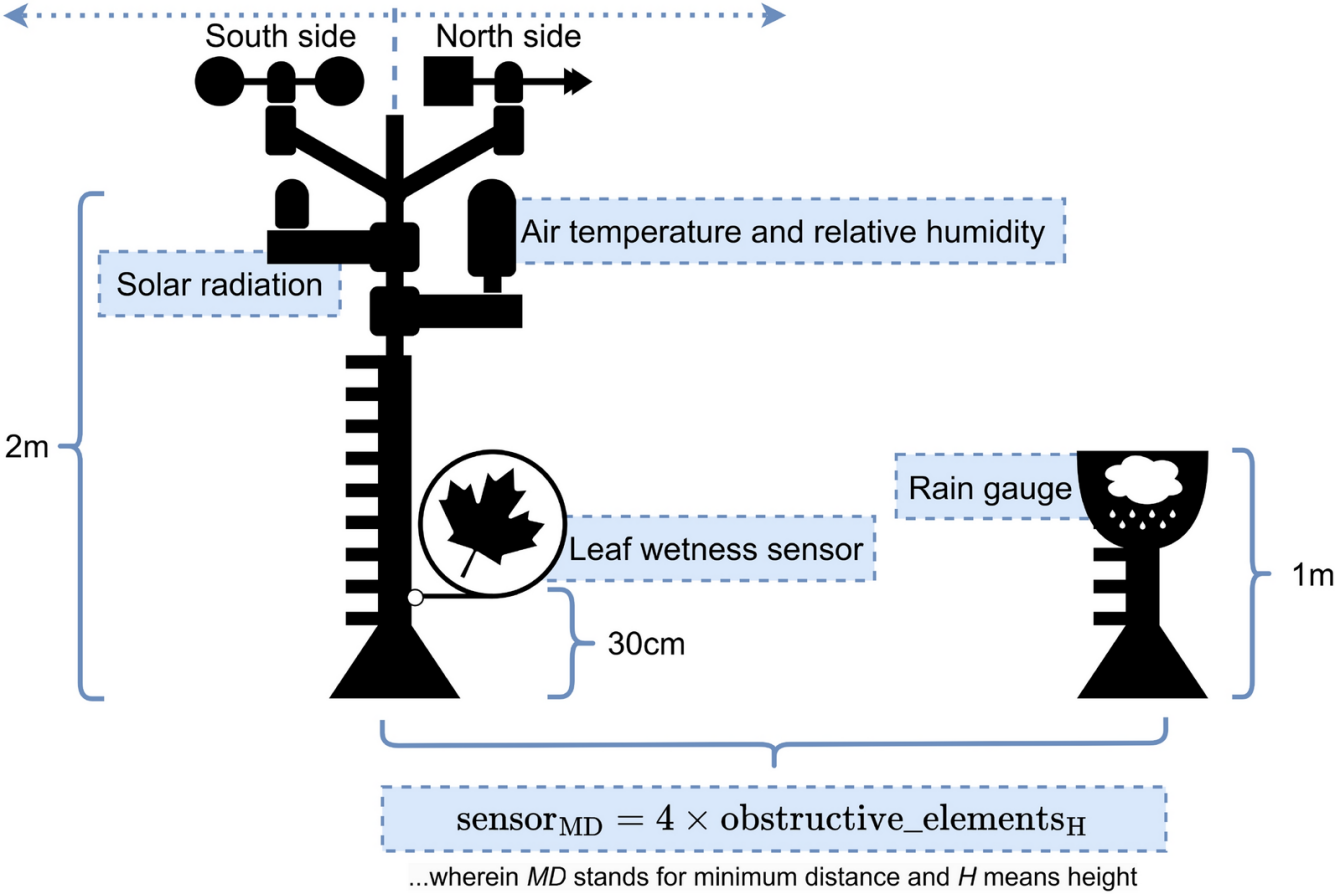
General guidelines for the installation of several sensors to monitor climate-related data, including solar radiation, air temperature and relative humidity, leaf wetness, and rain gauge sensors.

Figure 4 provides the primary installation guidelines for climate-related monitoring stations, which typically combine pluviometers with other sensors previously discussed, such as solar radiation, air temperature, humidity, and leaf wetness sensors.

### Cameras

In the scope of harmful insect detection, high-definition cameras are normally placed on top of gluing traps containing attraction pheromones, with the field of view aligned with the area of interest to monitor^141^. Regarding the placement of traps in the vineyards, it should be carried out at the grape clusters level and with a distance of, at least, 50 cm from the ground^210^. To monitor chromotropic traps, a high-definition camera should be used^141^, placed at 1.5 m high^211^. Cameras can also be placed directly within the vegetation canopy, pointing at an insect attractant such as, for example, sentinel cards consisting of European grapevine moth eggs and pupae placed at the level of the grape clusters^212^.

To monitor crops canopy, cameras should be placed on vertical poles with variable heights and angles, considering image quality, as well lightning conditions^152^. For example, Zorer^213^ recommends a minimum height of 2.5 m. Alternatively, Lloret et al. ^147^ reported satisfactory results by placing cameras with a small 360°rotating engine on top of 6-m-high poles.

### Microphones

Microphones should be placed on top of poles with a minimum height of 1.5 m^214^ distributed throughout the area. The number of microphones varies with the desired precision and the microphones used. The more microphones are placed, the greater the precision obtained and covered area. When considering microphones capable of listening in a range of 150–200 m, between 8 and 12 microphones are necessary to cover approximately 1 $\hbox {km}^2$ with acceptable precision, when using microphones that capture frequencies from 50 Hz to 20 kHz^153^.

Table 11 summarizes the key best practices for setting up and using all types of IoT-capable sensors above discussed.

**Table 11:** Summary of the recommendations and precautions to consider, per IoT-capable sensor type installation.

IoT sensor goal	Recommendations	Precautions
Soil water content and soil matric potential	Single application point should be representative of water retention characteristics for the whole terrain For grapevines, the application should be made in the area explored by the roots and the number of sensors used should represent the root zone’s total heterogeneity To monitor water waste and percolation depth, sensor should be placed at the bottom of the root.	An appropriate soil analysis should be done to increase the accuracy of the sensor Depending on the rootstock used, the depth should vary between 20 cm to 60 cm Depending on the rootstock used, the horizontal distance from the trunk where the sensors should be positioned may vary with the geotropic angle of roots.
Air temperature and relative humidity	A ventilated capsule with solar radiation protection should involve the sensor.	Nearby elements’ obstructive potential should be mitigated ($\mathrm {sensor_{MD}} = 4 \times \mathrm {obstructive\_elements_{H}}$, wherein *MD* stands for minimum distance and *H* means height) Sensors must be kept away from air temperature and humidity influencing factors (30 m of minimum distance).
Wind speed and direction	Installation should be made considering a minimum height of 2 m.	Nearby elements’ obstructive potential should be mitigated ($\mathrm {sensor_{MD}} = 10 \times \mathrm {obstructive\_elements_{H}}$, wherein *MD* stands for minimum distance and *H* means height).
Solar radiation	Installation should be made considering a maximum height of 3 m In the Northern Hemisphere, sensor should be installed on the south side of the main station.	Ensure installation far from shadow-inducing elements Ensure installation far from reflexive surfaces and artificial installation sources.
Atmospheric pressure	Installation should be made 1 m high.	It should be kept away from any type of obstruction.
Sap flow	Sensor installation should occur at night When using aluminum channels, the use of heat-conducting paste application is recommendable on sap flow sensor probes Aluminum channels should be sprayed with an antiseptic product before application Tape to fasten the sensor is advisable, preventing positional drifts and air entry in xylem vessels Protections against environmental influences are recommendable Sensors locations should be changed regularly.	Do not install in nodes or dead wood The depth of the xylem vessels in the trunk should be well known Holes sensor entry must be parallel to each other Before making holes in trunk, bark residuals must be cleaned out Heat method pulse requires 2 temperature sensors and a heating system probe, placed with specific distances from each other (5 and 10 mm) Garnier method requires 2 temperature sensors, one of them with a heating system probe incorporated, placed at 10 cm distant from each other.
Leaf temperature	The sensor should measure the temperature of leaves directly exposed to sunlight.	Foliage obstruction should be avoided.
Leaf wetness	The installation should be made 30 cm high An orientation of 45° should be given to the sensor In the Northern Hemisphere, sensor should be installed on the north side of the main station.	Close contact with artificial humidity inducers as irrigation systems should be avoided.
Stem Water Potential	To stabilize the readings, it is advisable to wait a couple of days after installation The sensor requires protection against environmental factors.	The depth of the xylem vessels in the trunk should be determined in advance This sensor must not be installed in nodes or dead wood Before making holes in trunk, bark residuals must be cleaned out.
Leaf Turgor	Leaves inside the canopy with an apparent good health status should be preferred to the attachment of the sensor Sensor requires calibration before use.	Measurements overlapping leaf veins must be avoided.
Dendrometer	For an effective binding of the device to the trunk, spring or cyanoacrylate glue can be used Support and wood portion in contact with the dendrometer require monitoring in terms of temperature fluctuations in an enhanced precision in measurements.	Installation should be made away of critical points such as ground or the morphological irregularities of the trunk Rhytidome should be removed for an effective contact between device and trunk It requires calibration before installation (resorting to a micrometer or metal plates) Exposure to sun and raindrops needs to be mitigated.
Reservoirs’ water level	It is advisable to install the sensor roughly in the center of reservoirs Placing the sensor inside a perforated tube is a good practice.	Installation should be done away from obstacles.
NPK, pH, and soil electrical conductivity	While the correct sensor installation depends on the plant species and despite the apparent scarcity of guidelines in literature, there are recommendations to take several terrain samples for laboratory analysis, allowing to assess the best spots for placing the measuring devices and increasing the accuracy of the sensor The number of sensors should represent the root zone’s total heterogeneity For grapevines, the application should be made in the area explored by the roots and the number of sensors used should represent the root zone’s total heterogeneity.	Depending on the rootstock used, the depth should vary between 20 cm to 60 cm Depending on the rootstock used, the horizontal distance from the trunk where the sensors should be positioned may vary with the geotropic angle of roots.
Soil oxygen	While guidelines for vineyard installation seem not to exist, for avocato orchards, there are proposals suggesting installations at a depth of 35 cm.	Installations guidelines may vary with the crop type and sensor specifications The horizontal distance from the trunk where the sensors should be positioned may vary with the geotropic angle of roots.
Soil temperature	Installation depth depends on the goals Typical installation involve burying the sensor 10cm bellow soil level Measurements that can better consider plant water status representation requires the sensor to be installed in the close peripheries of the respective root Suitable installations are made in a 1 Fflat surface in the center of 10 m radius circular area.	Installation should be made keeping, at least, 1.5 m of distance to edified structures Sensors should be kept as far as possible from natural runoff zones.
Rain gauge (pluviometer)	The installation should be made 1 m high Capture funnel should be oriented to the sky, with the minimum possible inclination relatively to the ground plane.	Nearby elements’ obstructive potential should be mitigated ($\mathrm {sensor_{MD}} = 4 \times \mathrm {obstructive\_elements_{H}}$, wherein *MD* stands for minimum distance and *H* means height).
Cameras	Installation distances and orientation depend on the purpose and camera resolution When used inside the delta traps, the delta traps should be around 50 cm of distance from the ground for maximum efficiency When used to monitor the chromotropic traps, the chromotropic trap should be around 1.5 m high for maximum efficiency Regarding crop monitoring, there are recommendations in literature pointing to installation in poles, at heights between 2.5 m and 6 m, wherein rotating engine may ensure an increased field-of-view coverage.	Cameras configuration require confirmation of the area in its field-of-view In a typically dusty environment, lens cleaning must be carried out from time to time.
Microphones	Microphones should be hung in poles heightening 1.5 m To cover 1 $\hbox {km}^2$, 8-12 microphones equidistantly placed are required.	Microphones need to be safeguarded from or have integrated resilience to ambient conditions (rain, sun heating, etc.) These sensors are not recommended for noisy environments.

The individual capabilities of sensors can be amplified through their combination, which has been a common practice in PV to complement or increase the range of available data regarding crops management, as shown in the following subsection.

### Practical examples on useful combinations of sensors for PV

With water management concerns in mind, King et al.^215^, proposed a DSS for vineyards that integrates the data of several sensors. More specifically, an IoT platform was set up, connecting sensors capable of measuring grapevine canopy temperature, soil water content, solar radiation, air temperature, relative humidity, and wind speed. The combination of these data provides an accurate assessment of the CWSI, replicating the methods proposed by Jackson et al.^50^. By combining both the CWSI with the soil water content data, the DSS was able to successfully automatize irrigation procedures in terms of both water needs and administration scheduling.

In a similar line of research, Jiménez et al.^216^ proposed a system that combines sensors to maximize water use efficiency, utilizing 25 nodes with various combinations of different sensor types. The sensors used allowed to assess soil water behavior based in water content and matric potential, and to evaluate plant tendencies according to water availability, through the monitoring of trunk diameter variation and canopy temperature. Only three nodes were used to collect climatic data (air temperature, relative humidity, vapor pressure deficit, and atmospheric pressure). This sensors combination allowed the system to precisely adjust irrigation to the grapevines’ water needs, considering environmental conditions. This multi-parameter irrigation management approach demonstrates an effective strategy for water administration by integrating information about the plant’s physiological state, soil water content, and climatic conditions surrounding the plant.

Mattedi et al.^217^ presented a methodology similar to the previous study, but focusing on continuous monitoring of plant water potential using FloraPulse microtensiometers installed directly in the grapevine trunks. To assess soil water dynamics, soil matric potential sensors were strategically positioned at 30 cm depth, under the drip line of the drip irrigation system. The monitoring of the vineyard’s surrounding environment was done through a weather station located 700 m away from the study site, from which air temperature, relative humidity, and solar radiation data was considered. This approach has proven effectiveness in managing precise irrigation based on soil, plant, and environmental data. The system applies specific volumes of water, optimizing the use of this resource while efficiently responding to the needs of the grapevines.

Bălăceanu et al.^16^ developed a comprehensive IoT platform capable of monitoring a wide range of parameters: climatic conditions (air temperature, relative humidity, atmospheric pressure, solar radiation, precipitation, wind speed and direction), leaf surface humidity, and soil conditions (soil temperature, soil water content, soil oxygen content, soil electrical conductivity, nutrient content). This platform offers an integral view of vineyard conditions, assisting in pest and disease prevention, nutrient monitoring, and irrigation management. The authors emphasize that this approach provides a comprehensive and detailed view of the conditions influencing the vineyard, with effective results in pest and disease prevention, nutritional monitoring of the soil around the plant roots, and irrigation control.

The study by Postolache et al.^103^ proposed a simple multi-sensor system for evaluating the plants surrounding environment. Using two types of low-cost sensors – one for environmental conditions (air temperature, relative humidity) and another for soil (soil water content, soil pH, soil temperature, soil electrical conductivity, soil nutrient content) – their system provides relevant data that can be used in several critical tasks such as smart irrigation strategies, fertilization, monitoring of some bioclimatic indices, and control over pests and diseases.

Another IoT station equipped with low-cost sensors to monitor and predict the evolution of vineyard diseases (Downy mildew, Powdery mildew, Black rot, and Botrytis) in real-time was implemented by Trilles et al.^218^, who integrated disease models that produce estimations when queried with specific weather conditions. The adopted sensors set includes air temperature, relative humidity, wind speed, precipitation, and soil temperature. Such data is collected and analyzed on an hourly basis, allowing for rapid alerts to viticulturists about potential infections. This approach not only improves response capabilities, but also promotes more sustainable viticultural practices by providing insights that can be considered to aid in the reduction of phytosanitary products usage.

Marcu et al.^219^ presented a study that, while similar to the previous example in its real-time weather monitoring approach, stands out for its efficiency with limited resources. The system uses only three sensors: air temperature, rain gauge, and solar radiation. Despite the simplicity of the setup, the authors demonstrate that it is possible to perform comprehensive analyses, including the assessment of the vineyard’s water regime, disease monitoring, and tracking of phenological cycles. This study exemplifies how a minimalist but strategic selection of sensors selection can provide valuable information for vineyard management.

A last example on sensors combination is inspired by the work of Kontogiannis et al.^220^, who developed a system that uses data sources characterizing environmental conditions and grapevines physiological states. More specifically, this work employs air temperature and relative humidity sensors, solar radiation sensors, leaf surface humidity sensors, soil water content sensors, and RGB cameras with infrared capabilities. The integration and processing of such multi-source data enables various practical applications in viticulture, such as irrigation management, disease prevention and management, canopy management assistance, plant stress monitoring, yield and quality prediction, climate prediction-based adjustments, zoning, and determination of optimal harvest time.

The presented examples shown that sensors fusion, when carried out based on specific physiological needs, are essential for the effectiveness of precision viticulture. The integration of data from sensors into IoT platforms allows for more informed, adaptive and time-effective management, promoting resource efficiency and sustainability in viticultural practices.

The next and last section briefly discusses the range of themes previously addressed for the array of sensors surveyed for PA/PV, followed by concluding remarks.

## Discussion and conclusions

After consulting the scientific literature related to close-range IoT sensors in the scope of PA/PV, the following application groups were identified: smart irrigation, detection of pests and diseases, smart fertilization, monitoring and mapping vegetation cover, and monitoring and tracking climate evolution. Moreover, climate and soil sensors, as well as cameras are among the most adopted devices for agricultural/viticultural management, under a monitoring perspective. While different sensors are applied to distinct monitoring strategies and purposes, the quality of the data obtained depends, not only on the accuracy of the sensor, but also on the procedures taken for their installation in the operational environments.

Precision irrigation through rigorous water management in viticulture is crucial for ensuring crops’ cost-effective quality—e.g. grapevines—, especially in a context of increasing demand and climate change^221^. Several techniques/methods have been proposed within this theme, focusing the monitoring of soil—e.g. soil matric potential^76,77^—, and plants’ biophysics—e.g. crop evapotranspiration, sap flow^76^ and leaf turgor^64^. Another noteworthy approach regards to water reservoirs instrumentation^78,79^, which is more exogenous to the plants’ organics but fairly relevant for assessing water availability in crops’ surrounding natural or man-made repositories. Regarding the advantages and disadvantages, they differ according to target. For example, methods built for soil are simple to install but the identification of a representative ground area is challenging. For the sensors that were designed to be in contact with the plant (trunk/leaves), the pros are more variable. When responsiveness is a requirement, sap flow is one of the candidates; clear readings (under ideal conditions) are more associated to leaf turgor. On the other hand, disadvantages related to calibration or repositioning needs are common to methods such as crop evapotranspiration, CWSI, or leaf turgor (for more information, revisit Table 2). Some known manufacturers of smart irrigation-capable sensors include Campbell Scientific, Decagon, Sentek, ICT International, Dynamax, Yara, Sensotec Instruments, among others (for more information, revisit Tables 8–10). As for the installation, sap flow is one of the more complex to set up. It requires, for example, tape to fasten the sensor and protections against external environmental factors, among several other special procedures to attach it to the plant. Leaf turgor requires avoiding main leaf veins for correct readings (for more information, revisit Table 11).

In the scope of smart fertilization, there are also various strategies oriented to decision-making with impact in crops-related productivity. Besides soil electrical conductivity^134,135^, and soil temperature^77^, diverse strategies for nutrients monitoring—e.g. electrical chemical and optical sensors—are available^133^. Regarding the latter class, in spite of developments towards low-cost, ease-of-use, portable or field-deployable sensors are still ongoing^221^, there are some already available for quantifying NPK, but still with many restrictions. Besides, for other macro and micronutrients, an affordable effective proximity option seems to still be lacking. In terms of disadvantages associated to nutrient sensors, the most noteworthy include the need for complex laboratory analysis and the variation of reliability at different soil depths. Regarding the advantages, while optical sensors are non-destructive, electrochemical- and soil electrical conductivity-based ones have good times of response (for more information, revisit Table 3). JXCT is one of the manufacturers that can be found in the market for providing NPK sensors (for more information, revisit Table 10). Recommendations and precautions during the installation of such sensors include to find suitable installation spots based in the laboratorial analysis of several soil samples and also to determine an adequate distance to the plant’s trunk considering its roots’ geotropic angle (for more information, revisit Table 11).

As for pests and diseases identification, while climate-based data offers insights to predict possible outbreaks with relatively reliable accuracy^150^, digital equipment specialized in collecting specific environmental elements, such as sound, can be used to inspect the presence of invasive species (e.g. birds) with potentially nefarious effects for the crops^143,153^. Within the digital acquisition equipment, imagery sensors are of major importance. More specifically, when RGB imagery is combined with artificial intelligence (AI), computational inference skills increase outstandingly, as shown in many works of the literature regarding pests identification and quantification^141,142,149^. On the other hand, and in spite of being able of providing massive amounts of data collected from hundreds of bands across the EM spectrum, hyperspectral cameras still have prohibitive costs, which has been limiting their use to science, mostly^222^. Notwithstanding, hyperspectral resolution has been demonstrating its pertinence in several tasks such as the early detection of pests and diseases, allowing to increase the effectiveness of phytosanitary treatments, reducing costs, and ensuring the quality of the alimentary feed-stock^223^. In terms of advantages, climatic data can provide large-scale areas characterization, while imagery and microphones are simple to install and can have a supporting system (e.g. an AI node) trained to provide several inference services (e.g. the classification of image or sound related to various crop-threatening species). Noteworthy disadvantages related to climatic data include the need of continuous and accurate historic. Moreover, while imagery sensors are usually sensitive to illumination conditions, microphones require a considerable scale-up of equipment (for more information, revisit Table 4). In terms of manufacturers, many of them can be found in both scientific and market contexts. Climate data usually resorts to a range of environmental indicators that can be provided by services as mySense^19^, or through a congregation of climate-related IoT sensors including air temperature sensors and pluviometers, which are provided by manufacturers in the line of Bosch or Vaisala. In case of imagery sensors, known providers include Basler Vision Technologies, BaySpec and Ocean Optics. Many manufacturers are also available as microphones providers, with examples of application like the one proposed by Nagy et al.^143^ (for more information, revisit Tables 9 and 10). Regarding the recommendations/precautions to install such sensors, each equipment contributing for a climate data-based methodology has specific guidelines. On the other hand, for cameras and microphones, there are distance and coverage-based recommendations that can be followed, along with precautions related to the environment wherein such devices are meant to be installed. For example, microphones do not operate well in noisy environments and cameras are prone to dust accumulation which may hamper image capturing as time goes by (for more information, revisit Table 11).

Due to their spectral and spatial flexibility in data acquisition, imagery sensors go beyond the identification of pests and diseases. Besides RGB sensors, other EM-responsive devices that may even exceed the visible EM spectrum, as it is the case of multispectral cameras, have been ringing the attention of the communities of PA/PV scientists and professionals due to their applicability and increasingly affordability^224^. In what regards to the monitoring and mapping of vegetation cover, RGB and multispectral cameras seem to be the most used imagery techniques. They have plenty of applications essential for understanding the distribution, vigor, and changes in crops, especially in vineyard plots, allowing for the identification of priority intervention areas and evaluation of actions effectiveness^166–174^. Nevertheless, comparatively to the UAV technologies, which are in constant proliferation due to their increased coverage capabilities, using stationary imagery sensors seems to be addressed at a shorter extent.

As for the climatic indices, they are fundamental to understand the insights characterizing climate evolution over time and its impact on crops^175^, as vineyards. With such insights, agriculture/viticulture professionals can optimize their procedures adequately and mitigate or even prevent the nefarious effects of climate variability in production. Several specific indices have been reported^176,179^, including the night cold index, dryness index, and various microclimatic indices, aiming to establish correlations between the environmental indicators of a given area and measurable biological factors in plants/fruits to evaluate the potential quality of a given location for cultivating or maintaining specific crops. Some hydrothermic indices can also provide insights to assess the risk of downy mildew proliferation—a direct contribution for pests and diseases identification. One of the most interesting characteristics of these methods is the ability to anticipate critical issues even before crops development season. Although, continuous data accuracy is required for the models to perform well. Several indicators—many of them common to climate data-based techniques formerly addressed in the subject inherent to pest and diseases identification—can be used to perform climate indices-based monitoring: soil water content, air temperature, air relative humidity, wind speed and direction, solar radiation, pluviosity and waterproof temperature (for more information, revisit Table 7). Among the precautions and recommendations to consider in the installation of the respective sensors include guidelines such as positional configurations with specific distance ranges relative to an environmental element (e.g. root, soil depth, ground-based height), orientation rules, obstructions avoidance, etc. (for more information, revisit Table 11).

Besides their individual use, one must keep in mind that combining sensor holds significant potential for the development of more effective PA/PV DSS, through the integration of diverse data sources. Several works^215–217^ have proven such potential for advanced smart irrigation, by merging a wide variety of sensors to monitor soil water content and matric potential, air temperature, relative humidity, atmospheric pressure, among other parameters. General crops status assessment was covered by other proposals^16^, wherein the integration of sensors specialized in measuring several climatic, leaf-based and soil conditions demonstrated effectiveness in disease prevention, nutritional monitoring and irrigation control. The sensor fusion proposed by Postolache et al.^103^ gathers critical data that may support in tasks such as smart irrigation and fertilization. Moreover, serious vineyard diseases can be closely monitored and predicted by considering the articulation of several weather-related sensors, as proposed by Trilles^218^. Marcu et al.^219^ used a similar set of sensors not only for diseases monitorting but also for tracking phenological cycles. Focusing some of the previous challenges, but also others such as yield and quality prediction an zoning, Kontogiannis et al.^220^ merged environmental conditions monitoring with grapevines physiological assessment. Indeed, all of these examples highlight the importance of strategically expanding available PA/PV-related data sources in a purpose-driven manner to broaden insights into crops—including vineyards—and to enhance critical tasks that impact both production and the environment.

As it is observable along this survey, PA/PV have shown significant advancements in close-range sensing technologies. A thorough categorization and characterization of IoT-capable sensors— including their respective pros and cons—applicable to agriculture, and, more specifically, to viticulture, highlights their crucial role in addressing production optimization challenges with environmental awareness in a context of significant climate variability. By including practical orientations for selecting and installing IoT-capable sensors across various monitoring applications, the present document aims to serve as a reference primarily for viticulture and agriculture professionals, as well as PA/PV practitioners. This audience should be able to interpret the recommendations and insights more effectively by following the steps outlined below:

- Identification of the application field(s) (e.g., smart irrigation, optimized fertilization);
- Alignment of a careful integration plan when combining multiple techniques/sensors, to meet the expected data articulation requirements and considering the due optimizations according to the available budget (e.g. complementarity and/or redundancy approaches);
- Selection of the desired sensing technique(s), considering the theoretical basis, effectiveness, advantages and limitations of each method (for example, within smart irrigation, a wide panoply of methods ranging from plants’ biophysics assessment to water reservoirs monitoring are available);
- Identification of the commercial sensors that can materialize a deployable solution (for example, within sap flow monitoring, off-the-shelf equipment may be acquired from sellers such as Dynamax, Greenspan or ICT International);
- Implementation following the best installation practices, to mitigate devices malfunctioning and ensure data reliability (for example, during the setting up a rain gauge, one must consider a minimum installation height of 1 m , while avoiding obstructive factors in its surroundings).

Such guidelines aim to empower the implementation of IoT systems in a DIY paradigm. However and in line with the recommendations underlined by this document, to undertake surveys of relevant manufacturers and suppliers towards the identification of the most suitable sensors for an IoT solution that best fits specific needs is highly encouraged, as technology evolves rapidly and market dynamics shift frequently.

## Data Availability

All data analysed during this study are included in this published article.
