# Author Correction: A technical survey on practical applications and guidelines for IoT sensors in precision agriculture and viticulture

**DOI:** 10.1038/s41598-025-90404-6

**Published:** 2025-02-28

**Authors:** David Pascoal, Nuno Silva, Telmo Adão, Rui Diogo Lopes, Emanuel Peres, Raul Morais

**Affiliations:** 1 Department of Engineering, School of Sciences and Technology, University of Trás-os-Montes e Alto Douro, Quinta de Prados – Folhadela, 5000-801 Vila Real, Portugal; 2 Centre for the Research and Technology of Agro-Environmental and Biological Sciences, University of Trás-os-Montes e Alto Douro, 5000-801 Vila Real, Portugal; 3 ALGORITMI Research Center/LASI, University of Minho, Alameda da Universidade, 4800-058 Braga, Guimarães, Portugal; 4 Institute for Innovation, Capacity Building and Sustainability of Agri-Food Production, University of Trás-os-Montes e Alto Douro, 5000-801 Vila Real, Portugal

Correction to: *Scientific Reports* 10.1038/s41598-024-80924-y, published online 30 November 2024

The Funding section in the original version of this Article was omitted. The Funding section now reads:

“This work is funding by the Vine and Wine Portugal Project, co-financed by the RRP–Recovery and Resilience Plan and the European Next Generation EU Funds, within the scope of the Mobilizing Agendas for Reindustrialization, under the reference C644866286-00000011.”

Additionally, the acknowledgements were incorrect.

“Finally, this research activity was co-supported by national funds from the FCT-Portuguese Foundation for Science and Technology under the projects UIDB/04033/2020 and LA/P/0126/2020.”

Now reads:

“Authors would like to acknowledge from the FCT-Portuguese Foundation for Science and Technology under the projects UIDB/04033/2020 and LA/P/0126/2020.”

The original Article has been corrected.

